# Solution-Deposited Ferroelectric BiFeO_3_ Perovskite-Based Films: A Spotlight on Their Manifold Applications in Emerging Technologies

**DOI:** 10.1021/acsaem.4c02906

**Published:** 2025-02-27

**Authors:** María Lourdes Calzada, Iñigo Bretos, Ricardo Jiménez, Jesús Ricote, Rafael Sirera, Miguel Algueró, Adriana Barreto, Yadira Andrea Rivas, María Echániz-Cíntora

**Affiliations:** † Instituto de Ciencia de Materiales de Madrid, Consejo Superior de Investigaciones Científicas (ICMM-CSIC), C/Sor Juana Inés de la Cruz, 3. Cantoblanco, 28049 Madrid, Spain; ‡ Departamento de Química, Universidad de Navarra, C/Irunlarrea, 1, 31008 Pamplona, Spain

**Keywords:** BiFeO_3_ perovskite films, chemical solution deposition (CSD), multiferroic, magnetoelectric, photovoltaic, photocatalysis

## Abstract

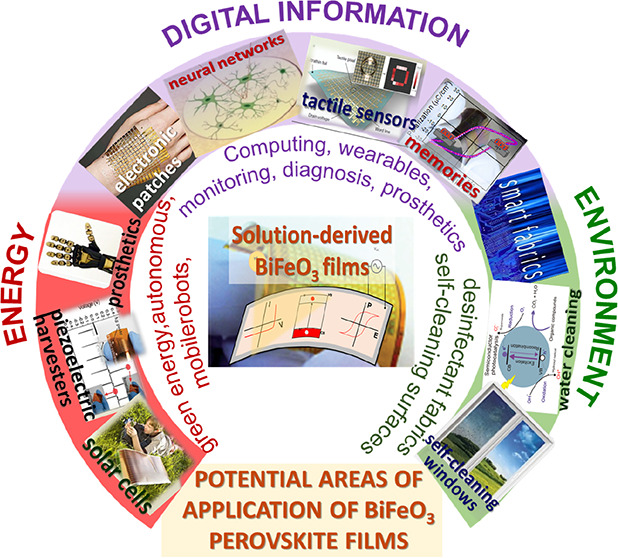

The advancement of smart materials is crucial for addressing the cross-cutting challenges of contemporary society. These materials are expected to help raise living standards through the expansion of smart cities, efficient management of natural resources, pollution control, and improvements in social welfare. Consequently, the multifunctionality of ferroelectric oxides makes them ideal candidates for meeting these demands. Among ferroelectric oxide materials, bismuth ferrite (BiFeO_3_) stands out as a multiferroic compound with ferroelectric, ferroelastic, and antiferromagnetic properties at room temperature. It also has one of the lowest bandgaps among ferroelectrics, making it a photoferroelectric compound with both photovoltaic and photocatalytic properties. These responses can be fine-tuned by partially substituting Fe^3+^ ions with selected cations or by creating solid solutions between BiFeO_3_ and other ferroelectric perovskites. BiFeO_3_-based thin-film materials are regarded as ideal for harnessing the diverse properties of BiFeO_3_ in emerging technologies. Chemical solution deposition methods facilitate the design of crystallization pathways for metal oxides, such as BiFeO_3_ thin films, making them essential for developing low-temperature strategies that offer benefits ranging from reduced environmental impact to lower manufacturing costs. A greater challenge lies in preparing BiFeO_3_ films at temperatures compatible with their direct integration into flexible systems using polymeric substrates. This spotlight article highlights, through examples from our group’s research over the past decade, the various applications of BiFeO_3_-based perovskite thin films in emerging technologies. Interest is not only in devices based on rigid single-crystal substrates, like silicon, but also in those using flexible polymer substrates. Here, we discuss the promising opportunities of using low-cost, high-throughput solution deposition methods for producing multifunctional BiFeO_3_-based perovskite films for future applications.

## 1 Introduction

Bismuth ferrite (BiFeO_3_) exhibits a rhombohedral phase (*R*3*c* space group) at room temperature, with lattice parameters of *a* = 3.965 Å and α = 89.4° in a perovskite pseudocubic unit cell. The ferroelectricity of BiFeO_3_ perovskite originates from the interaction of Bi 6s^2^ lone electron pairs with the O 2p orbital, resulting in off-centered Bi^3+^ ions. BiFeO_3_ was revealed as a robust ferroelectric in the last century, with a high Curie temperature (*T*_c_) of 1103 K.^1^

The highest spontaneous polarization (Ps) of approximately 100 μC cm^–2^ among lead-free ferroelectrics was estimated from atomic coordinate calculations. However, the experimentally measured polarizations were 1 order of magnitude lower than the calculated values due to the challenges of obtaining high-quality BiFeO_3_ perovskite materials.^2,3^

At the beginning of this century, a large ferroelectric polarization of approximately 60 μC cm^–2^ was demonstrated in epitaxial BiFeO_3_ thin films grown on SrTiO_3_ substrates,^3^ and latter also in high-resistivity single-crystals.^4^

This breakthrough opened a new era in the research of BiFeO_3_ ferroelectric materials. Additionally, BiFeO_3_ features Fe–O–Fe bonds, which are the origin of its (anti)ferromagnetism, sustaining a cycloidal spin structure with a period of ∼62 nm below the Néel temperature of 643 K. These findings positioned BiFeO_3_ as the most competitive compound among single-phase multiferroics.^5^

**Figure 1:**
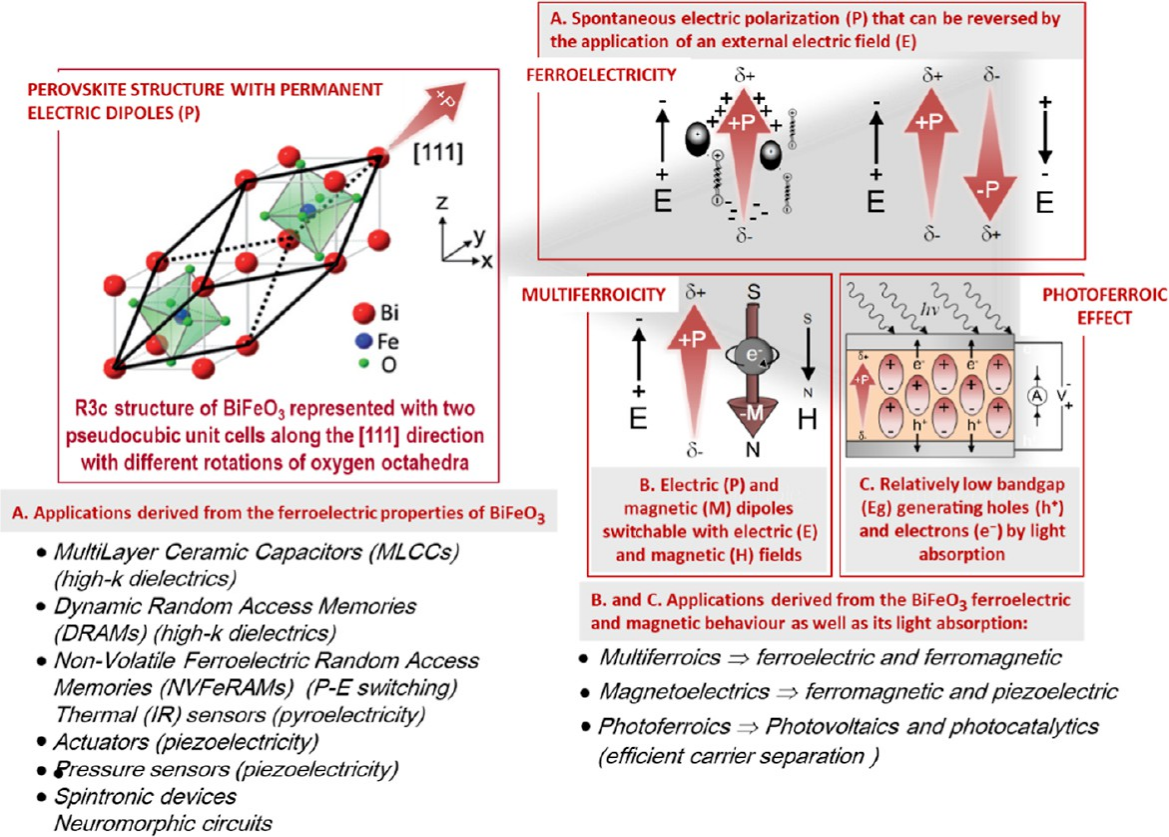
Crystal structure of bismuth ferrite (BiFeO_3_) perovskite is presented, illustrating the process of poling under an electric field, along with its multiferroic characteristics and the photoferroic effect in BiFeO_3_ perovskite materials. Applications derived from these physical properties, which are of interest for devices, are also highlighted.

Initially, research on BiFeO_3_-based materials focused primarily on their ferroelectric and magnetic behaviors, as these properties could be advantageous in various (micro-) electronic devices (Figure 1). However, the discovery that coupling between these properties could induce new physical effects, such as electric field control of magnetism and vice versa, and its high Curie temperature (*T*_C_), has expanded interest in other properties of BiFeO_3_, such as its magnetoelectric and piezoelectric responses.^5^ Moreover, the BiFeO_3_ perovskite has a bandgap of *E*_g_ ∼2.8 eV, one of the lowest among ferroelectric oxides. This low bandgap, coupled with ferroelectric polarization, enables BiFeO_3_ perovskite materials to exhibit relatively large photovoltages among ferroelectric oxides, resulting from the efficient separation of charge carriers by the ferroelectric polarized regions.^6^

The use of the polarization field to enhance charge carrier transfer and suppress recombination has led to polarization-enhanced photovoltaic and photocatalytic activity, garnering significant attention for BiFeO_3_-based perovskites in applications related to energy and environment, areas that were previously unimagined for BiFeO_3_ materials.^7−12^

**Figure 2:**
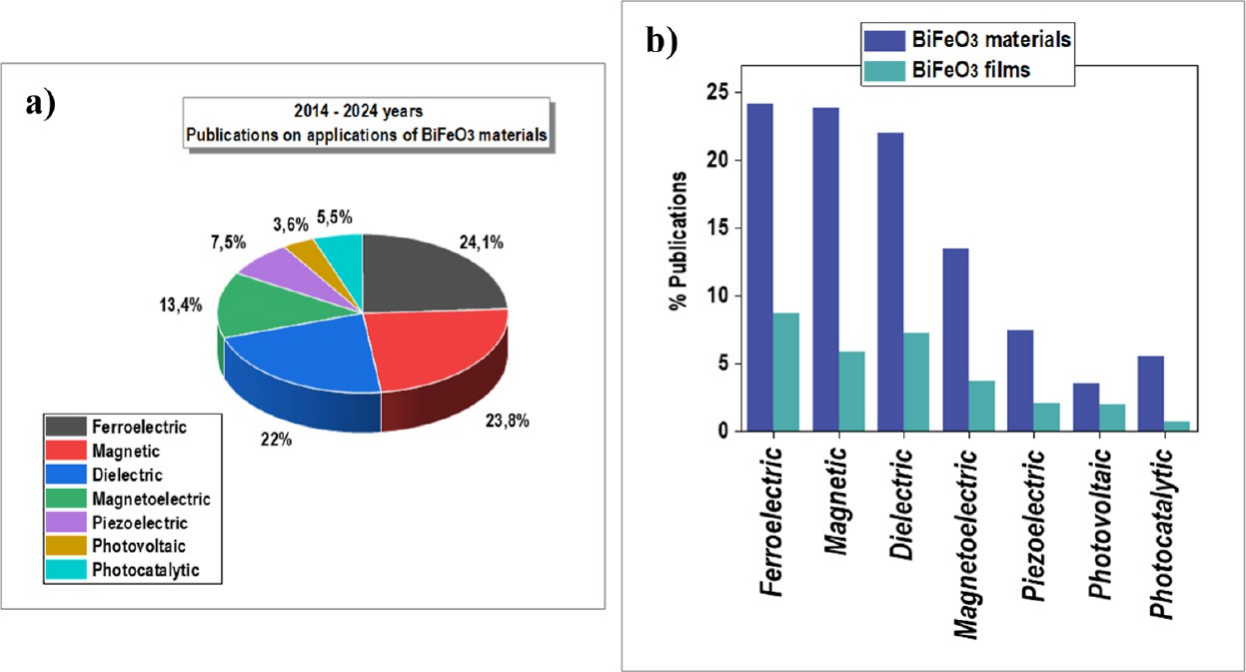
(a) Statistics of refereed publications on the properties of BiFeO_3_ perovskite materials useful for device applications over the past ten years, from 2014 to 2024. The total number of indexed publications on BiFeO_3_-based perovskite materials during this period is 16.826. (b) Statistics from the period 2014–2024 show a comparison of total publications on BiFeO_3_ materials and those specifically related to BiFeO_3_ films. The total number of indexed publications on BiFeO_3_-based perovskite films during this period is 5.111. Data collected from Web of Science.

Figure 2 shows statistics over the past ten years of refereed publications on BiFeO_3_ perovskite materials with properties useful in various application fields.

The fabrication of BiFeO_3_ materials in thin-film form is essential in the current semiconductor industry, as it facilitates the integration of functional materials into miniaturized devices for applications that are otherwise not feasible with bulk materials.^8,9,13−16^ These applications include ferroelectric memories, next-generation spintronic and neuromorphic devices, as well as piezoelectric, sensor, energy-related, and environment-related devices (Figure 1). However, the required characteristics of ferroelectric oxide films vary depending on the application. High-quality thin-film samples (mostly epitaxial films) are necessary to meet the stringent physical property requirements for memories, spintronic devices, neuromorphic systems, sensors, and actuators. This typically requires the use of rigid single-crystal substrates that promote the epitaxial growth of the film.

In contrast, large-area coatings, with less stringent quality requirements, are needed for photovoltaic applications to achieve high light-to-electricity conversion. A similar need arises for photocatalysts. In this field, powdered materials are typically used for photocatalytic remediation, which are dispersed in the medium to be treated, such as wastewater, but which must be removed after purification. This approach can lead to secondary pollution due to residual photocatalysts in the purified medium, and their removal increases the cost of the process. This issue could be addressed by supporting the catalyst as a thin layer on a substrate. Currently, BiFeO_3_ photocatalysts are considered a promising alternative to more traditional compounds like TiO_2_ anatase. The photocatalytic behavior of BiFeO_3_-based perovskite films is being explored for applications beyond contaminant remediation, such as solar-to-chemical conversion [e.g., hydrogen (H_2_) or methanol (CH_3_OH) production]. In this context, achieving high conversion efficiencies and low-cost processes also requires the fabrication of large-area coatings on inexpensive substrates.

Various deposition techniques have been used to fabricate BiFeO_3_ thin films, including pulsed laser deposition (PLD), radio frequency (RF) magnetron sputtering, chemical vapor deposition (CVD), and chemical-solution deposition (CSD).^3,17−20^

High-quality, dense BiFeO_3_ films with low defect content, minimal leakage, and reduced conduction have been produced using methods such as PLD, RF magnetron sputtering, and CVD.^17−21^ These deposition techniques enable the fabrication of ultrathin epitaxial films ideal for low-voltage applications, where devices also require high integration densities and excellent physical properties to deliver high performance. However, these methods are expensive, requiring high-vacuum equipment and complex facilities. This limits their use in consumer electronics, which increasingly demand affordable and smart devices to meet societal needs.

Chemical solution deposition (CSD) is cited in the literature as one of the most appropriate technique for fabricating these devices. The drawbacks of this technique associated with the toxicity and teratogenicity of the reagents used for synthesizing the precursor solutions have been significantly reduced in recent years due to the development of environmentally friendly solution strategies and low-carbon footprint processing methods.^22−26^ Consequently, CSD has been well-developed as an effective method for producing numerous functional thin films, offering significant advantages over other techniques, such as low cost, large-scale deposition, stoichiometric control, and uniformity. Furthermore, CSD provides opportunities that other deposition methods cannot offer.^27,28^ A major advantage is the ability to tailor the chemistry of the precursors to control the film’s microstructure and texture, as well as to tune its physical properties. In this scenario, reducing the processing temperature by designing the chemistry of the precursor solution is an achievement that makes the integration of the film with temperature-sensitive substrates more accessible.

**Figure 3:**
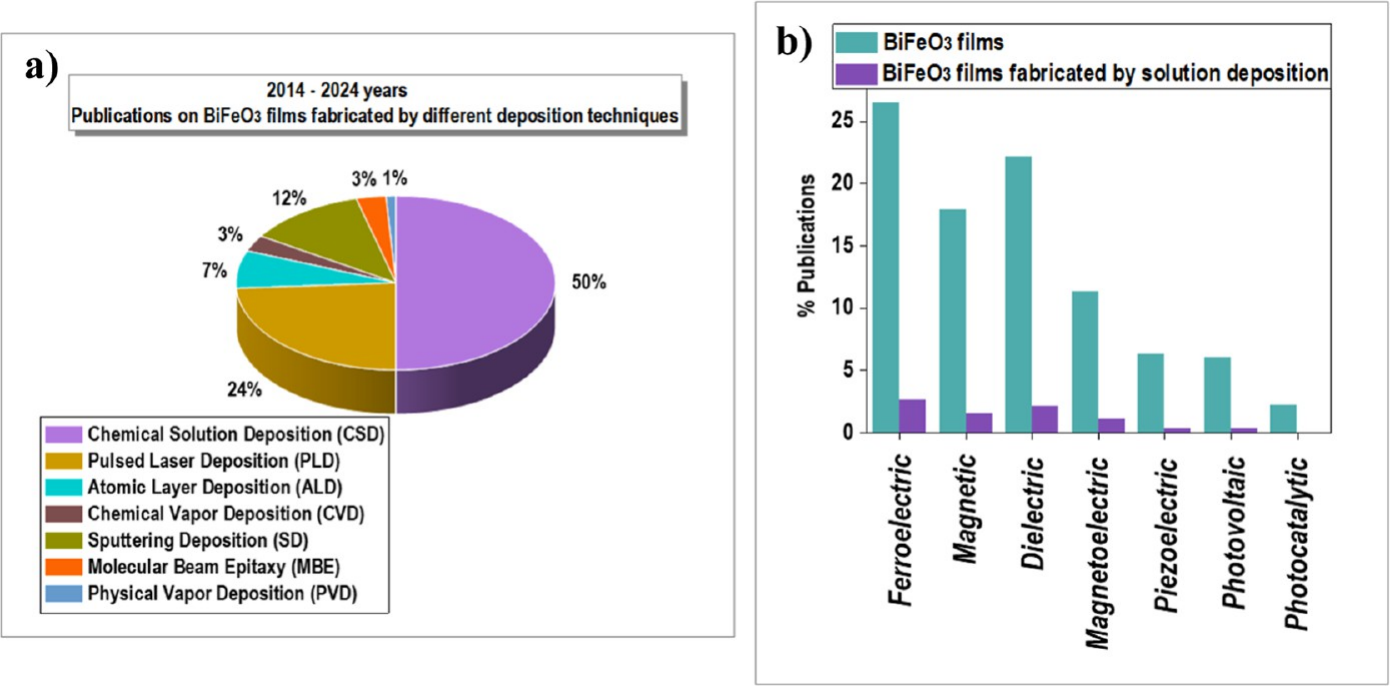
(a) Statistics from the period 2014–2024 show publications on BiFeO_3_ films fabricated by the most used film deposition techniques. (b) Statistics from the period 2014–2024 show publications on BiFeO_3_ films deposited by nonsolution techniques and BiFeO_3_ films fabricated by chemical solution deposition (CSD). Data collected from Web of Science.

**Figure 4:**
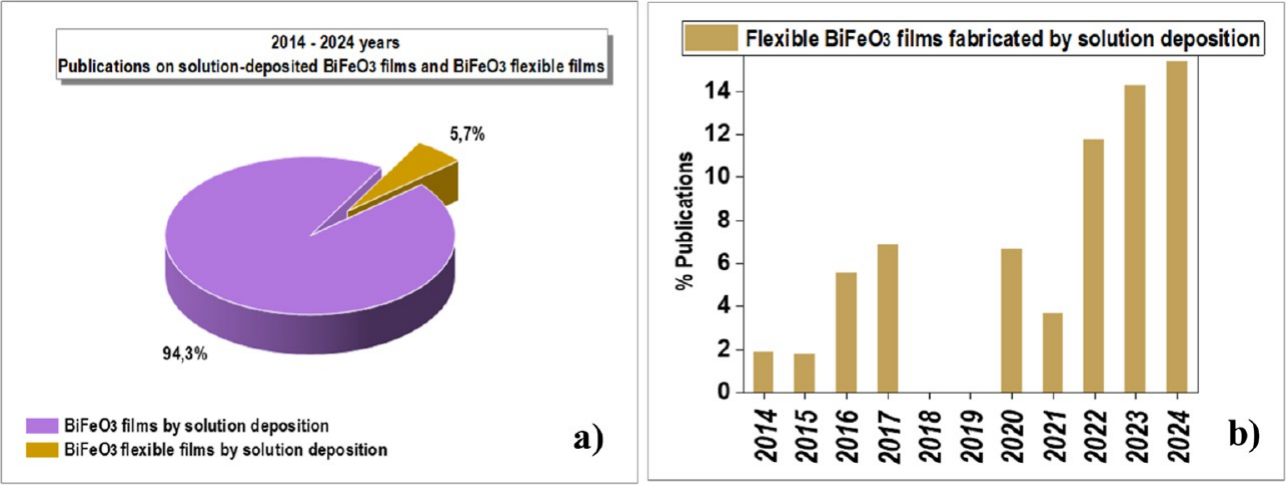
(a) Statistics from 2014 to 2024 showing refereed publications on BiFeO_3_-based perovskite films fabricated via chemical solution deposition (CSD), with a focus on publications related to flexible BiFeO_3_-based perovskite films obtained through CSD. (b) The percentage of publications on BiFeO_3_-based perovskite films directly fabricated on flexible substrates using CSD, calculated in relation to the total publications on BiFeO_3_-based perovskite films fabricated by CSD. Data collected from Web of Science.

Figure 3a presents statistics from the last ten years regarding publications on BiFeO_3_ thin films fabricated by different deposition techniques. It is clear that CSD is the most used method to attain BiFeO_3_ perovskite-based films. Focusing solely on the applications, Figure 3b shows a comparison of total publications on BiFeO_3_ films and those specifically related to BiFeO_3_ films fabricated by CSD in the different areas of applications of these films. The analysis of these publications reveals significant progress in the solution deposition of these perovskite oxide films, including epitaxial and highly textured films with properties similar to those of single-crystal-like BiFeO_3_ films achieved through costly and less feasible deposition techniques. In addition, CSD has enabled the fabrication of BiFeO_3_ films not only on conventional single-crystal substrates (Si, SrTiO_3_, or YAlO_3_) but also on flexible, temperature-sensitive substrates (metal foils or plastics). Figure 4a shows the number of publications on solution-derived flexible BiFeO_3_ films, specifically highlighting those where direct deposition on a large-area flexible substrate is achieved using low-temperature solution deposition methods. Note that the percentage of flexible BiFeO_3_-based perovskite films directly fabricated by solution deposition methods during this period is low, especially when compared with flexible solution-deposited films of other metal oxide compounds. This category includes amorphous metal oxide transistors, which are currently the most widely used materials in flexible electronics. However, Figure 4b highlights the progressive growth in publications on flexible BiFeO_3_-based perovskite films fabricated by CSD. Publications not included in these statistics pertain to flexible BiFeO_3_ films primarily fabricated using indirect deposition methods, where films are first sintered at high temperatures on rigid single-crystal substrates and later transferred to temperature-sensitive substrates. These indirect methods are costly and generally do not meet the requirements for most flexible devices, including photovoltaic and photocatalytic systems, as well as consumer electronics, where large-area devices are required.

In this Spotlight article, we provide a comprehensive overview of the applications of BiFeO_3_-based perovskite films fabricated using CSD methods. We summarize our group’s work over the past decade on solution-derived BiFeO_3_ thin films with thicknesses ranging from approximately 50 to 300 nm. We examine the results for films deposited on both rigid single-crystal substrates and flexible plastic substrates. Initially, interest in BiFeO_3_ films was driven by their unique characteristics as a single-phase, lead-free multiferroic with high ferroelectric polarization and room-temperature ferromagnetism. Fundamental studies on these properties highlighted their potential for a broad range of microelectronic applications that include advanced device concepts beyond conventional ones, such as memristive memories and neuromorphic systems. Additionally, with the advent of flexible electronics, ferroelectric perovskite films emerged as strong candidates for consumer electronics, offering an alternative to amorphous semiconductor oxides.

A key challenge has been reducing the high crystallization temperature of ferroelectric perovskite films, as ferroelectricity in these materials is inherently tied to their perovskite crystal structure. Recent advancements in low-temperature solution deposition methods have enabled the direct deposition of ferroelectric perovskites on flexible polymer substrates. This has opened new application areas, particularly for BiFeO_3_ perovskite, which has one of the lowest bandgaps among ferroelectrics. This discovery has expanded the possibilities for BiFeO_3_-based films beyond traditional microelectronic devices, advancing into postsilicon technologies in previously underexplored areas. Here, we discuss potential applications of solution-derived BiFeO_3_-based perovskite films in these emerging technologies, illustrated through examples from our group’s work.

## 2 BiFeO_3_ Perovskite and Film Solution Processing

Before the publication of the Materials 2030 Manifesto,^29^ the classical definition of advanced materials focused on those that exhibit unique or enhanced properties. In this sense, BiFeO_3_-based perovskite materials were clearly included in this category due to their intrinsic multifunctionality. However, The Materials 2030 Roadmap introduced the additional goal of maximizing the sustainability of these now redefined advanced materials. According to the Sustainable Development Goals,^30^ advanced materials should also meet two main criteria: the first is the use of compositions free from hazardous and critical elements, and the second is the adoption of environmentally safe fabrication processes.

BiFeO_3_ is a lead-free perovskite oxide traditionally considered an alternative to lead-containing ferroelectric materials. Its nontoxic composition includes bismuth (Bi), which is significantly less toxic than other heavy metals, enabling a variety of medical and industrial applications (e.g., alloys, pigments, electronic components, and fishing sinkers).^31^ However, Bi is classified as a critical raw material due to its low natural abundance (2 × 10^–5^ %), being obtained primarily as a byproduct in the production of copper, lead, and tin.^31^ Additionally, bismuth is part of the Technology-Critical Elements (TCEs), essential for the integrity of various industrial ecosystems and critical in manufacturing components for new technological and energy-related applications.

The European Commission first defined Bi as a critical and strategic raw material in 2017, a designation also recognized by the U.S. Department of the Interior (US DOI) in 2018.^32−34^ Given the strategic importance of Bi, there is a growing need to develop separation technologies for recovering bismuth from industrial, particularly electronic, waste,^35^ which is a crucial step for incorporating BiFeO_3_-based film devices in emerging technologies. In this context, BiFeO_3_ is regarded as a sustainable material.

**Figure 5:**
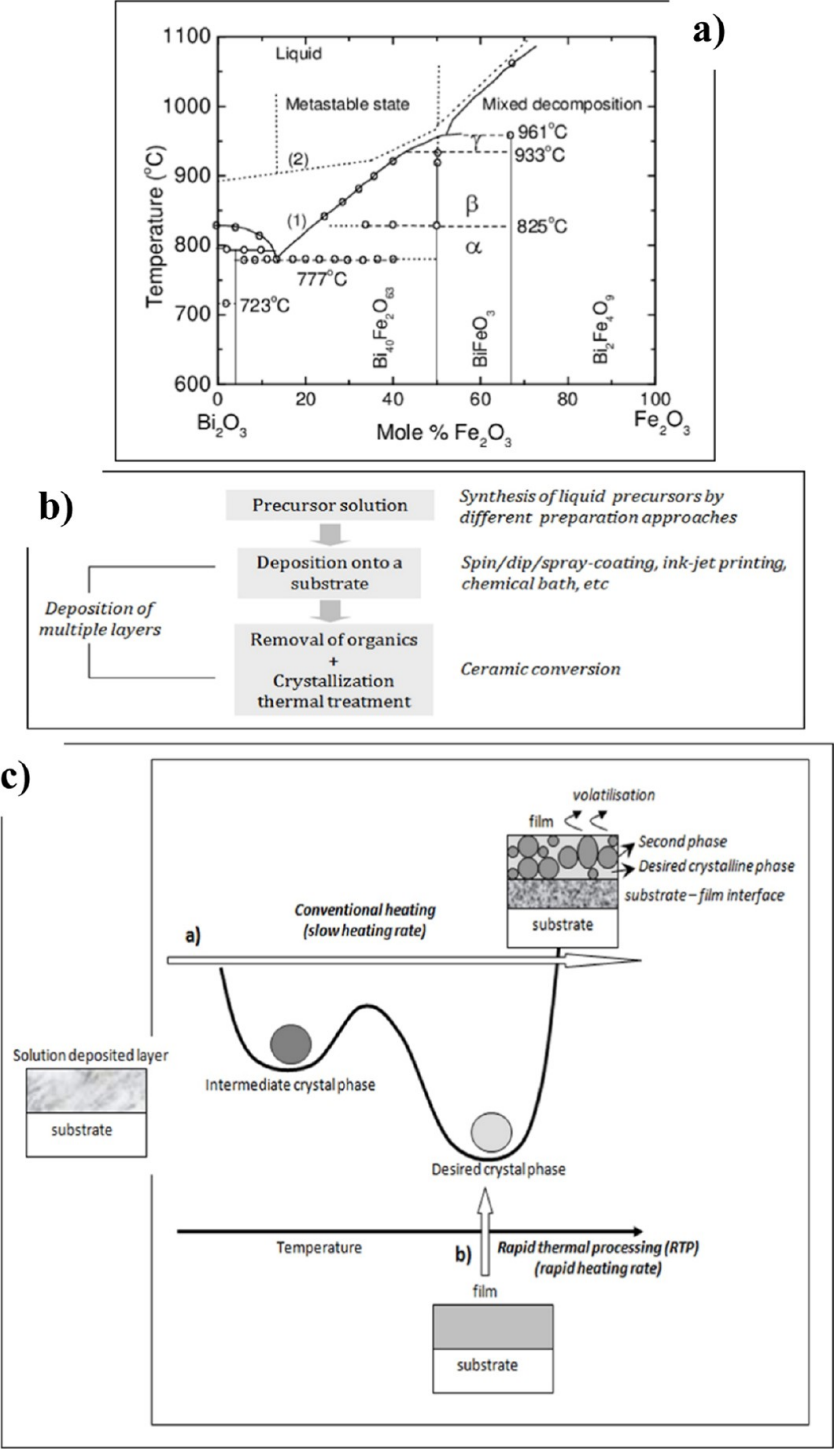
(a) Phase diagram of the Bi_2_O_3_-Fe_2_O_3_ solid solution, showing that the BiFeO_3_ perovskite is stabilized between 447 and 767 °C. It is flanked by bismuth-rich (Bi_25_FeO_39_) and iron-rich (Bi_2_Fe_4_O_9_) stable compounds. (b) Scheme illustrating the general process used for the deposition of films by chemical solution deposition (CSD). Film thickness is controlled by the precursor solution’s concentration and the number of solution layers deposited. (c) Comparison of the crystallization of solution-derived films using conventional heating and rapid thermal processing (RTP). The formation of single perovskite films is favored by RTP, as it rapidly passes through the temperatures where second phases are stable, minimizing substrate–film diffusion and avoiding the volatilization of volatile elements.

However, synthesizing BiFeO_3_ in its pure phase is challenging. It exists as a metastable phase between 447 and 767 °C, flanked by bismuth-rich (Bi_25_FeO_39_) and iron-rich (Bi_2_Fe_4_O_9_) stable compounds in the phase diagram (Figure 5a).^36^

Solid-state synthesis has been employed to crystallize BiFeO_3_ powders at temperatures above 700 °C.^37^ However, impurities in starting materials have significantly impacted the formation of the pure BiFeO_3_ perovskite phase.

Solution-based routes utilizing high-purity chemical reagents have proven effective for synthesizing phase-pure BiFeO_3_ powders at lower temperatures than solid-state methods. For thin films, the literature reports the use of CSD to obtain BiFeO_3_ films, employing various chemicals, solvents, and crystallization conditions. The synthesis of BiFeO_3_ precursor solutions typically involves common cation reagents, including bismuth(III) and iron(III) nitrates, dissolved in carboxylic acids or, traditionally, in the highly hazardous 2-methoxyethanol (C_3_H_8_O_2_) solvent used in sol–gel alkoxide chemistry.^18,38,39^ Wet layers are deposited from these precursor solutions to form amorphous layers, which require thermal treatment to crystallize the oxide film (Figure 5b).

The crystallization of solution-derived BiFeO_3_ films is influenced by the local atmosphere, affecting phase purity and the density of point defects within the film. Rapid thermal processing (RTP) has also been shown to impact the formation of single-phase BiFeO_3_ perovskite by rapidly passing through the temperatures at which secondary phases are stable, minimizing bismuth volatilization and reducing diffusion reactions between the film and substrate (Figure 5c).

### 2.1 Solution-Deposited BiFeO_3_–Based Perovskite Films on Rigid Single-Crystal Substrates

**Figure 6:**
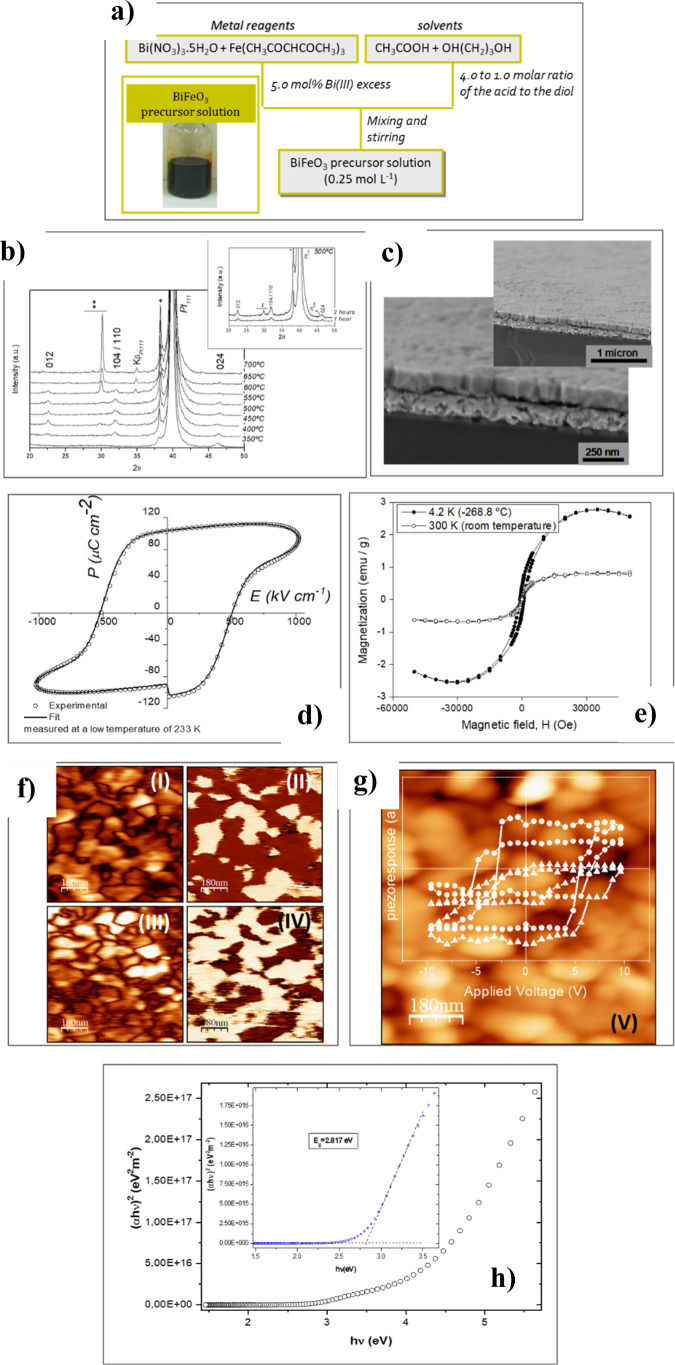
(a) Scheme of the processing of the BiFeO_3_ precursor solution. (b) X-ray diffraction (XRD) patterns of one-single layer film (∼40 nm thick) deposited from the BiFeO_3_ precursor solution and rapid thermal processed (RTP) at different temperatures for 1 h in air atmosphere. Films are onto Pt-coated (100) silicon substrates. The inset shows the XRD patterns of RTP films treated at 500 °C for different times. * Substrate, ** Bi_2_Fe_4_O_9_ phase. (c) Scanning electron microscopy (SEM) images of five-layer BiFeO_3_ films (∼200 nm thick) onto Pt-coated (100) silicon substrates. (d) Polarization vs electric field (*P*–*E*) ferroelectric hysteresis loop of the ∼200 nm thick BiFeO_3_ film annealed at 500 °C in air. Measurements were carried out at a frequency of 1 kHz and a temperature of −40 °C. (e) Magnetic hysteresis loops (*M*–*H*) measured for a ∼40 nm thick BiFeO_3_ film annealed at 500 °C in air. *M*–*H* loops were obtained in a superconducting quantum interface device (SQUID) magnetometer, applying a magnetic field parallel to the film surface. (f) Images of the out-of-plane piezoresponse force microscopy (PFM) amplitude and phase and in-plane PFM amplitude and phase. (g) Topography image of the BiFeO_3_ film. Remnant local piezoelectric hysteresis loops measured in different zones of this film are inserted in the topography image. (h) Direct band gap energy of *E*_g_ = 2.817 eV is graphically calculated from the inset of the figure. Adapted with permission from ref (18). Copyright © 2013, John Wiley and Sons.

Figure 6a shows the synthesis scheme used for the preparation of BiFeO_3_ precursor solutions, employing nontoxic reagents: bismuth(III) nitrate and iron(III) 2,4-pentanedionate, along with acetic acid and 1,3-propanediol as solvents.^18^Figure 6b displays the X-ray diffraction (XRD) patterns of a ∼40 nm thick film deposited from the precursor solution onto Pt-coated (100) silicon substrates, which were treated using Rapid thermal processing (RTP) at different temperatures for 1 h in an air atmosphere. Reflections corresponding to the formation of single-phase BiFeO_3_ perovskite are observed at temperatures between 400 and 550 °C. At higher temperatures, accelerated decomposition of BiFeO_3_ is indicated by the peak at 2θ ∼29°, which corresponds to the region where major reflections of iron-rich crystalline phases (e.g., Bi_2_Fe_4_O_9_) are recorded.^40^ Increasing the soaking time for RTP treatments at 500 °C in air also shows the progressive decomposition of the BiFeO_3_ films (see inset of Figure 6b).

The high quality and uniformity of these BiFeO_3_ films can be inferred from the scanning electron microscopy (SEM) images of a ∼215 nm thick BiFeO_3_ film shown in Figure 6c. Columnar growth is observed in these films, with an average lateral grain size ranging from 225 to 325 nm. Due to the high Curie temperature (*T*_c_) and consequently large coercive field (*E*_c_) of the BiFeO_3_ perovskite, high electric fields are required for the poling of these films. Furthermore, the initial switching of the ferroelectric polarization is hindered as domains are pinned by point defects, which are not oblivious to the challenge of achieving single-phase and stoichiometric BiFeO_3_ films. Consequently, BiFeO_3_ films often exhibit large leakage currents and conductivity at room temperature (RT), which hinders their performance. As a result, well-defined ferroelectric hysteresis (*P*–*E*) loops cannot be observed. As shown in Figure 6d, efficient switching of the ferroelectric polarization can be performed at low temperatures (∼233 K), at which nonswitching contributions are minimized, resulting in high values of remnant polarization, *P*_R_ ∼ 60 μC cm^–2^.

In addition to the strong ferroelectricity of these solution-derived BiFeO_3_ films, they also exhibit clear ferromagnetism (Figure 6e). Magnetic hysteresis (M-H) loops measured in ∼40 nm thick BiFeO_3_ films show ferromagnetism with noticeable remanence and coercivity. As expected, the magnetization decreases with temperature. Measurements of *M–E* loops in films of varying thickness reveal that the ultrathin BiFeO_3_ films (∼40 nm thick) possess significantly higher saturation magnetization values than thicker films (e.g., the ∼215 nm thick film shown in Figure 6c). Magnetization values at RT of ∼0.80 emu g^–1^ were measured for the ∼40 nm thick ultrathin films, which are comparable to those reported for epitaxial BiFeO_3_ films on SrTiO_3_ substrates. The epitaxial constraints between the film and substrate enhance magnetization due to the disruption of the ideal cycloidal spin structure. In these solution-derived BiFeO_3_ ultrathin films, the small grain size contributes to the interruption of the continuity of the cycloidal arrangements in both in-plane and out-of-plane directions, as the grain size in these directions is smaller than the ∼62 nm period of the cycloidal spin structure below the Néel temperature (*T*_N_).

Figure 6f presents the piezoresponse force microscopy (PFM) images of these BiFeO_3_ films. This PFM study indicates that the small grain size limits the formation of ferroelastic domains separated by twinning planes. This is characteristic of solution-derived polycrystalline thin films, which typically exhibit a grain size limited by film thickness. Nevertheless, the small crystal size does not diminish their ferro-piezoelectric properties, as evidenced by local piezoelectric loops (Figure 6g) and PFM images that demonstrate the piezoelectricity of these BiFeO_3_ films.

Finally, the data plotted in Figure 6h estimates a direct band gap energy of *E*_g_ ∼2.82 eV, which falls within the visible range (439 nm). This is consistent with values reported for phase-pure epitaxial BiFeO_3_ films and bulk single crystals. The optical band gap of this ferroelectric perovskite is close to the visible range, unlike most oxide ferroelectrics, such as LiNbO_3_, BaTiO_3_, or Pb(Zr,Ti)O_3_, which have higher band gaps (*E*_g_ ∼3.50 eV, ∼354 nm, falling into the ultraviolet range). In the early 2000s, these findings in BiFeO_3_ films expanded the potential applications of these materials, not only as multiferroics (ferroelectric and ferromagnetic) or piezoelectrics but also as photoferroics for use in photovoltaic or photocatalytic applications.

**Figure 7:**
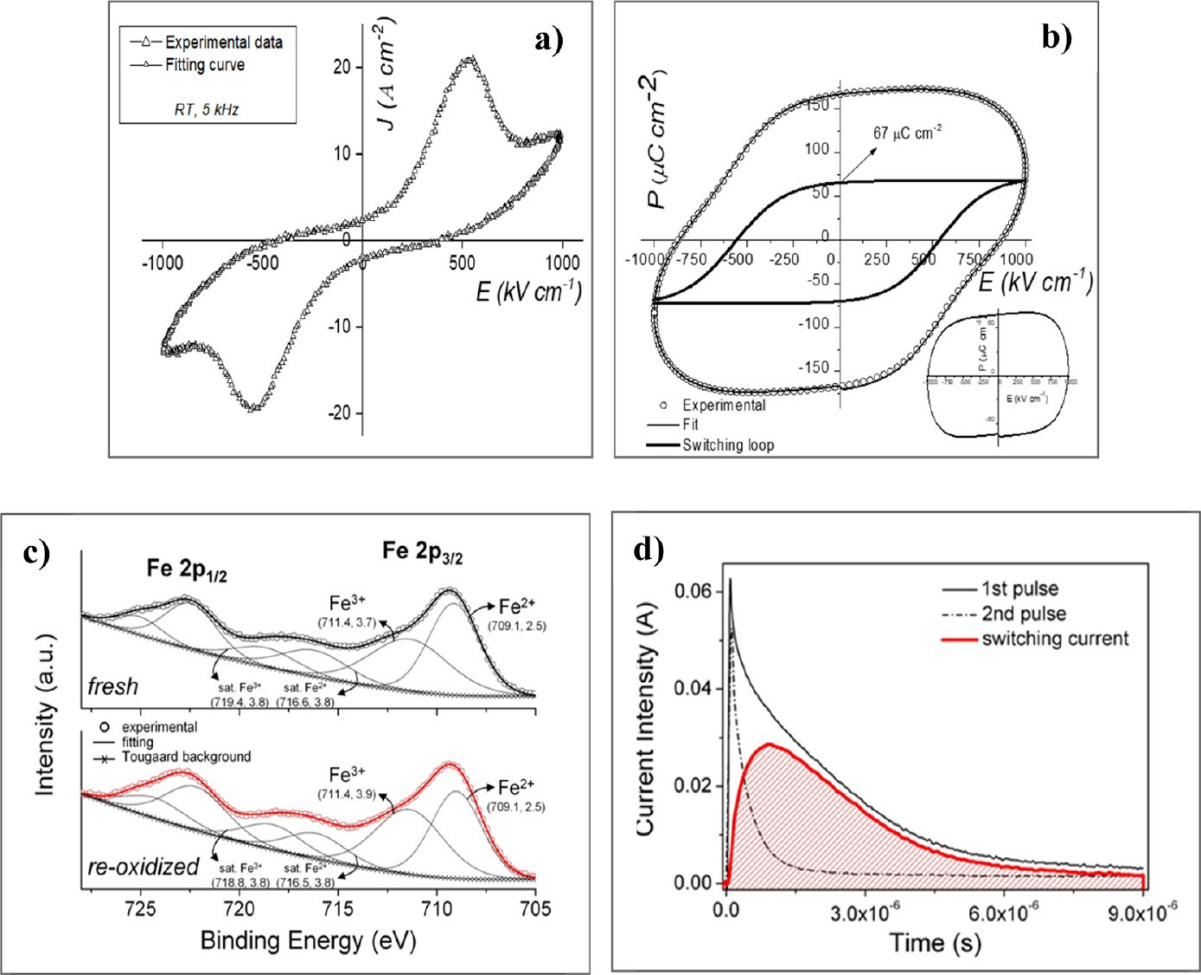
(a,b) Ferroelectric behavior of a solution-derived polycrystalline ∼215 nm thick BiFeO_3_ film. Current density vs electric field (*J*–*E*) curves and the corresponding polarization vs electric field (*P*–*E*) loops calculated from the integration of the *J*–*E* curves, are shown for measurements carried out at room temperature (RT). (c) Fe 2p core levels X-ray photoelectron spectroscopy (XPS) spectra showing in brackets peak maximum and FWDH width (eV) of fitting curves (sat. = satellite). XPS spectra of the film obtained after the RTP crystallization at 500 °C (fresh) and the called reoxidized film that was subjected to a further reoxidation annealing at 300 °C in a pure oxygen atmosphere. The existence of Fe^2+^ in the films agrees with the plausible formation of the Fe^2+^–VO^••^ defect dipoles, which hinder domain switching during the poling of the film at RT. (d) Switching current density measured at 233 K in the ∼215 nm thick BiFeO_3_ film before and after the reoxidation process. In this curve, P* and *P̂* are calculated from the measurements with the first (first) and second (second) pulse, respectively. The dashed area corresponds to the difference between P* and *P̂*, which corresponds to the pure ferroelectric switched polarization, Δ*P* = 2*P*nv. Adapted with permission from ref (41). Copyright 2014, AIP Publishing.

The presence of charged defects, primarily associated with Fe^2+^ ions and oxygen vacancies (O^••^), is identified as the main factor contributing to the relatively high leakage currents and conductivity in BiFeO_3_ thin films.^41^ Besides, these defects can pin ferroelectric domains, hindering their switching. Figure 7a,b show the current density vs. applied electric field (*J*–*E*) curve and the ferroelectric hysteresis (*P*–*E*) loop measured at room temperature for the ∼215 nm-thick BiFeO_3_ film. The *P*–*E* loop is obtained by integrating the experimentally measured *J*–*E* curve, which shows high conductivity and leakage characteristics, as indicated by the curve’s shape. This results in the leaky loop seen in Figure 7b. Comparing this loop with that shown in Figure 6d, measured at a lower temperature of ∼233 K, demonstrates that low temperatures reduce the mobility of the charged defects under an electric field, thus minimizing nonswitching contributions to the loop and revealing ferroelectricity. Consequently, when measurements are performed at a low temperature of 233 K, the charged defects freeze, allowing ferroelectric switching to be disclosed.

To assess the presence of charged defects in these BiFeO_3_ films, X-ray photoelectron spectroscopy (XPS) analysis was conducted on this film, along with XPS analysis of the same film after a reoxidation process, which involved heating the BiFeO_3_ perovskite film at 300 °C in an oxygen atmosphere. The XPS spectrum fitting analysis (Figure 7c) indicates the coexistence of both Fe^3+^ (711.4 eV) and Fe^2+^ (709.1 eV) states in both BiFeO_3_ samples, attributable to the common valence fluctuation of this ion. However, the Fe^3+^/Fe^2+^ ratio calculated from integrating the fitting curves at the Fe 2p_3/2_ level (including satellite peaks) increases in the reoxidized film (from 0.809 to 1.041). This suggests a relative increase of ∼6.3% in Fe^3+^ concentration following the dissociation of the Fe^2+^–VO^••^ complex defect present in the film before reoxidation. Consequently, the reoxidized film demonstrates an improved ferroelectric response, as confirmed by the pulsed polarization characteristics shown in Figure 7d. Integrating the switching current transients between two consecutive read pulses in the poled reoxidized sample yields a pulsed, nonvolatile polarization of 110 μC/cm^2^ (2P_R_), one of the highest values reported for thin films of this multiferroic composition.

**Figure 8:**
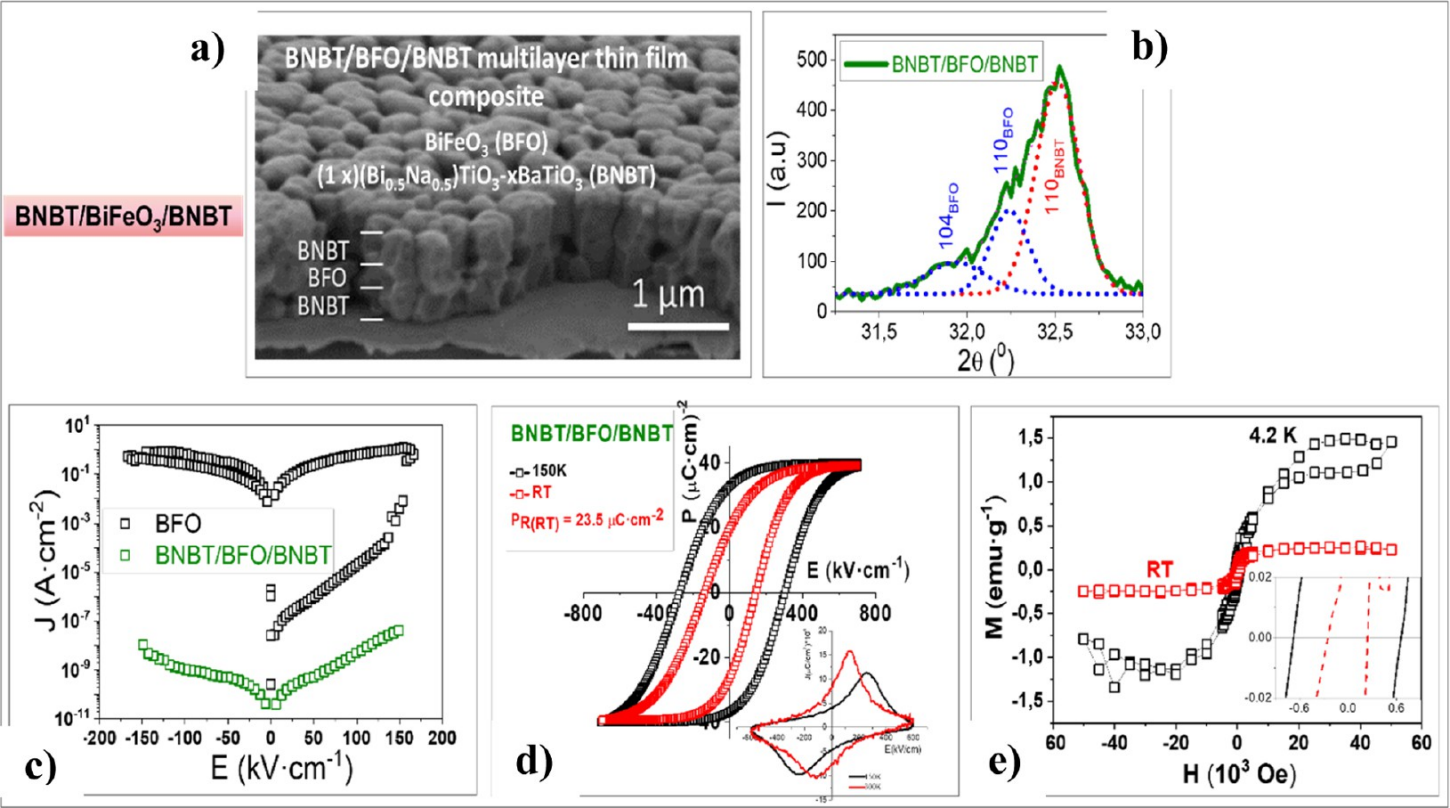
(a) Scanning electron microscopy (SEM) of the cross-section of the (Bi_0.5_Na_0.5_)_1–*x*_TiO_3_-Ba_*x*_TiO_3_/BiFeO_3_/(Bi_0.5_Na_0.5_)_1–*x*_TiO_3_-Ba_*x*_TiO_3_ (BNBT/BFO/BNBT) multilayer composite film. (b) X-ray diffraction (XRD) pattern of the BNBT/BFO/BNBT multilayer composite, showing the deconvolution of experimental diffraction peaks. (c) Leakage currents measured for the single phase BiFeO_3_ and for the BNBT/BFO/BNBT multilayer composite film. (d) Corrected *P*–*E* hysteresis loops measured at RT and low temperature (150 K). The inset shows the experimental *J*–*E* curves measured at RT and 150 K. (e) Magnetic hysteresis loops (M-H) measured at RT and low temperature in the BNBT/BFO/BNBT multilayer composite film. Adapted with permission from ref (42). Copyright 2015, AIP Publishing.

Our group developed a novel approach to reduce leakage currents in BiFeO_3_ thin films and to attain functional properties suitable for devices. This approach combines the relaxor/ferroelectric (Bi_0.5_Na_0.5_)_1–*x*_TiO_3_–Ba_*x*_TiO_3_ (BNBT) with BiFeO_3_ (BFO) layers in a multilayer composite film, as shown in the cross-sectional image in Figure 8a.^42^ This image also demonstrates the characteristic columnar growth of BiFeO_3_ films, previously observed, now replicated in the multilayer composite film. In this design, the BiFeO_3_ film is sandwiched between two BNBT layers, preventing direct contact between the BiFeO_3_ films and the Pt electrodes of the capacitor device, including both the Pt-bottom electrode onto the silicon substrate and the Pt-top electrodes on the surface of the multilayer composite film. Figure 8b illustrates the presence of the two crystalline phases in the multilayer composite film, originating from the individual layers, suggesting that significant interdiffusion does not occur between the alternating BNBT and BiFeO_3_ layers.

As previously demonstrated (see Figure 7), the large leakage currents and conductivity of BiFeO_3_ films at room temperature are associated with point defects that introduce free charge carriers in BiFeO_3_, which hinder the application of the electric fields needed to switch the material’s large ferroelectric polarization. We have shown that this behavior can be mitigated through the combination with other ferroelectric/relaxor layers that increase the electric field directly applied to the BiFeO_3_ layer. These in-series layers act as a sink for the point defects moving across the BiFeO_3_ film. As shown in Figure 8c, these trapping layers surrounding the BiFeO_3_ film serve as an effective interfacial engineering strategy to significantly reduce the leakage currents of the BiFeO_3_ film, as demonstrated in Figure 8d. Consequently, a reduction in the apparent coercive field (*E*_c_) of the film is attained, which allows polarization switching to occur at room temperature (Figure 8d). As illustrated, controlling leakage currents in BiFeO_3_-based films is essential for their application in ferro-piezoelectric systems as well as in magnetoelectric devices.

Figure 8e presents the M-H hysteresis loops of these multilayer composite films (thickness ∼120 nm) at room and low temperatures (RT and 4.2 K). Both loops indicate magnetic behavior with clear remanence and coercivity, and with values of the saturation magnetization (*M*_S_) of approximately 3.3 emu g^–1^ at 4.2 K and 1.1 emu g^–1^ at room temperature. These magnetization values are similar to those measured in single-phase BiFeO_3_ films of 40 nm thickness, suggesting that the columnar growth likely induces a constraint in the film comparable to the epitaxial constraints observed in epitaxial BiFeO_3_ films, which, combined with the size effects of solution-derived polycrystalline BiFeO_3_, releases latent magnetization.

**Figure 9:**
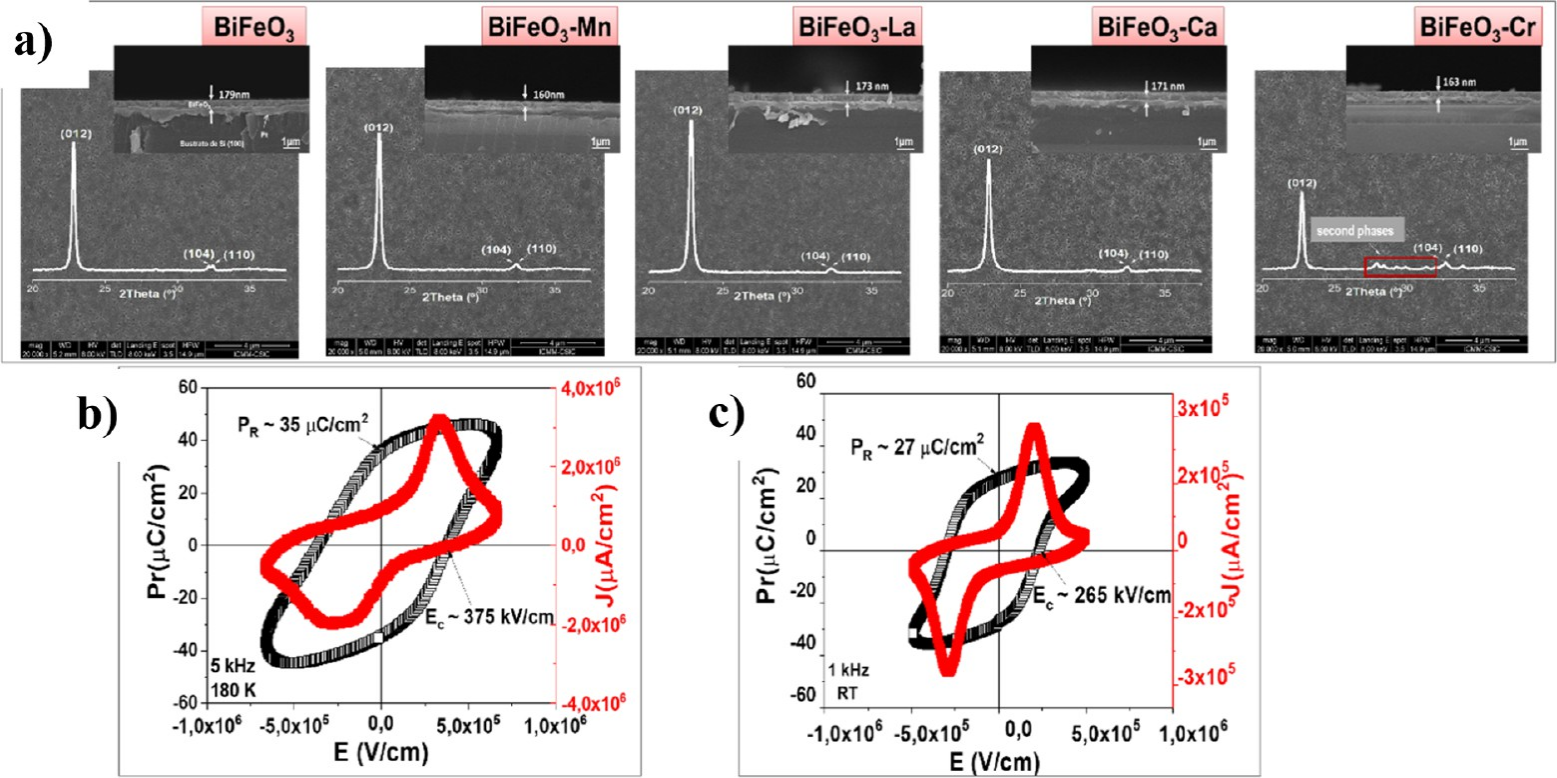
(a) Surface and cross-section scanning electron microscopy (SEM) images of BiFeO_3_ thin films doped with different cations (single-phase BiFeO_3_ and doped with manganese, lanthanum, calcium and chromium). (b,c) *P*–*E* hysteresis loop and *J*–*E* curves of the pure BiFeO_3_ and Mn-doped BiFeO_3_ films, respectively. The pure BiFeO_3_ films have been poled at a low temperature of 180 K and the Mn-doped BiFeO_3_ films have been poled at RT.

An alternative approach to reducing the content of charged defects in BiFeO_3_ thin films and thereby enhancing their ferroelectric response at room temperature is through cation substitutions at the Bisite, Fe-site, or both within the perovskite structure.^43^Figure 9a shows the SEM images (surface and cross-section) along with the XRD patterns of BiFeO_3_ thin films where calcium or lanthanum has been substituted at the B-site and manganese or chromium at the Fe-site, with a 5 mol % concentration of the dopant cation. No significant differences are observed in the films’ microstructures or in the XRD patterns, all of which show a preferred orientation along the ⟨012⟩ direction of the rhombohedral perovskite, typical of BiFeO_3_ films on silicon substrates. However, it should be noted that Cr-doped BiFeO_3_ films exhibit an appreciable content of secondary phases, which is likely due to the known difficulty of incorporating chromium into this perovskite structure.

Figure 9b,c show the *P*–*E* ferroelectric hysteresis loop and *J*–*E* curves of pure BiFeO_3_ and Mn-doped BiFeO_3_ films, respectively. These measurements indicate that the content of charged defects has significantly decreased with the manganese doping, as it reduces the coercive field (*E*_C_), allowing polarization at room temperature (RT). In contrast, poling of pure BiFeO_3_ films must be performed at a lower temperature (180 K) to achieve an effective ferroelectric response. In addition, doping BiFeO_3_ thin films with these cations also reduces their bandgap (*E*_g_), enhancing their photovoltaic response. Photovoltaic ferroelectric devices could autonomously power themselves with little maintenance, and at the same time, could operate with multiple functions (e.g., memories, sensors or actuators). Therefore, BiFeO_3_-based ferroelectric perovskites are foreseen as ideal candidates for self-powered devices.^6,44^

An effective application of these compounds as solar absorbers has been demonstrated for BiFeO_3_ perovskites with significant substitution of iron by chromium at the B-site of the ABO_3_ perovskite, forming the BiFe_1–*x*_Cr_*x*_O_3_ double perovskite; a solid solution of BiFeO_3_ and BiCrO_3_. BiFeO_3_ and BiCrO_3_ have bandgaps of approximately 2.8 and 1.4 eV, respectively, and maximum theoretically calculated values of spontaneous polarization, *P*_S_, of around 95 and 67 μC cm^–2^, respectively.^45^ However, a major issue with this double perovskite is that it exists as a metastable high-pressure (HP) phase, stabilized only in powder form through high-pressure and high-temperature synthesis.

In thin-film form, BiFe_0.5_Cr_0.5_O_3_ perovskite has been stabilized on SrTiO_3_ single-crystal substrates using pulsed laser deposition (PLD) with rigorously optimized parameters.^7^ The SrTiO_3_ substrates impose compressive stress on the BiFe_0.5_Cr_0.5_O_3_ film, promoting epitaxial growth. This epitaxial strain encourages the incorporation of chromium into the perovskite structure, thereby stabilizing the metastable BiFe_0.5_Cr_0.5_O_3_ phase. Additionally, it favors Fe^2+^/Cr^4+^ cationic ordering in the perovskite film, which helps to reduce the bandgap (*E*_g_) of the film. Consequently, solar cells with a power conversion efficiency of approximately 3.3% have been fabricated, one of the highest efficiency obtained to date for a photovoltaic device utilizing a ferroelectric oxide material as the sole absorber.^7^ Although these efficiencies are lower than those achieved with hybrid halide perovskites, ferroelectric oxide perovskites offer important advantages, such as high stability in harsh environments (e.g., resistance to temperature, moisture, chemicals, or radiation). However, it should be taken into account in the particular case of the BiFe_1–*x*_Cr_*x*_O_3_ double perovskites, the toxicity of chromium, which would limit the use of these compounds in sustainable functional devices.

**Figure 10:**
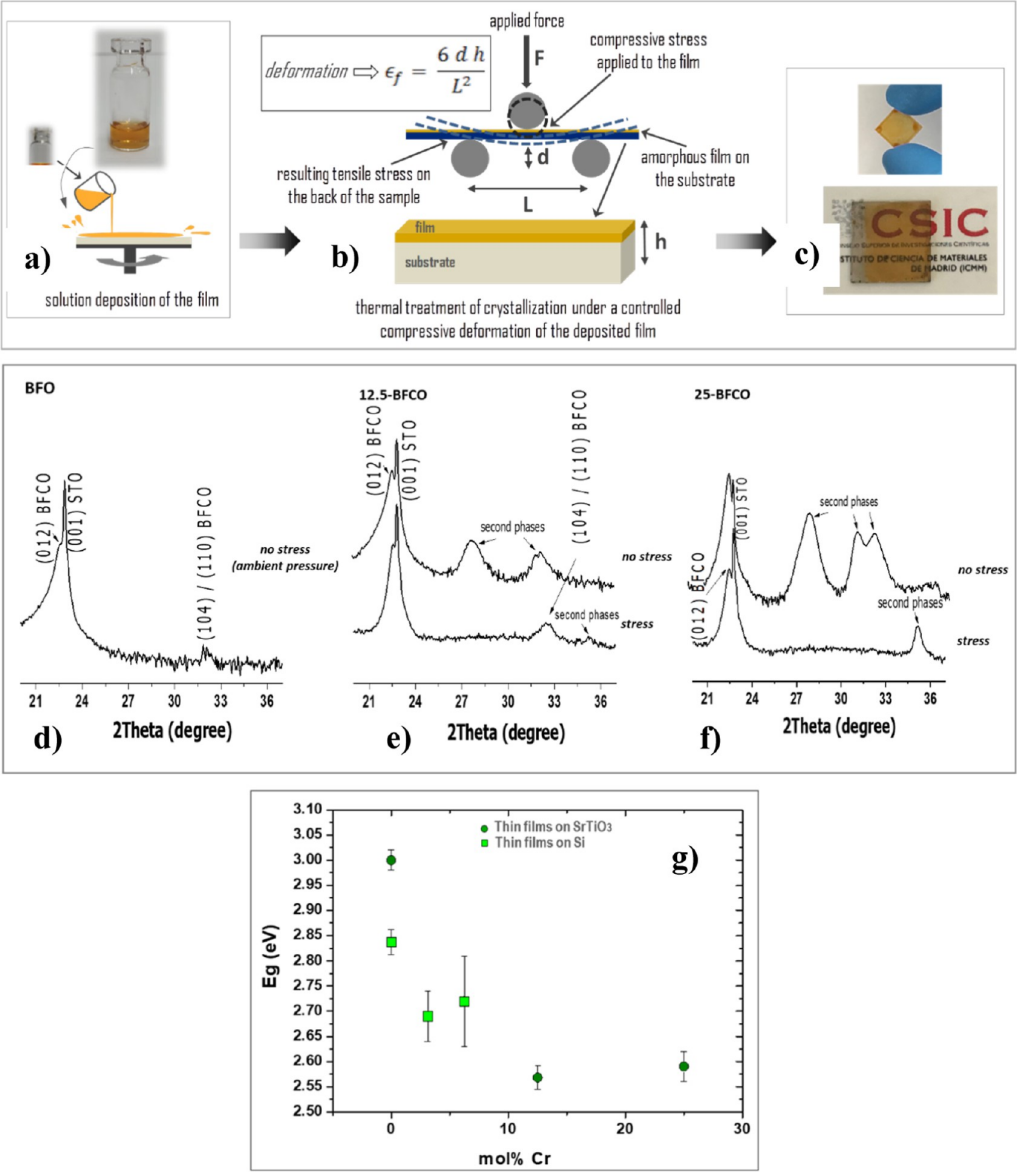
Scheme of the stress-mediated chemical solution deposition (CSD) method used to induce the crystallization and stabilization in thin film form of high-pressure (HP) metastable double-perovskite phases, in which a controlled force is applied to the solution deposited film during the thermal treatment of crystallization of the film. (a) The film is spin-coated from the precursor solution. (b) The deposited amorphous layer is subject to a controlled compressive deformation during the thermal treatment of crystallization. The formula inserted is used to calculate the deformation of the film. In this formula €_f_ = deformation, *d* = height displacement of the sample, *h* = sample thickness (film + substrate) and *L* = sample length in between the support bars. (c) Photographs of the HP metastable double-perovskite oxide thin films obtained under these boundary processing conditions on SrTiO_3_ substrates. X-ray diffraction (XRD) patters of ∼50 nm thick BiFe_1–*x*_Cr_*x*_O_3_ (BFCO) films on SrTiO_3_ (STO) substrates, with (d) *x* = 0 mol % of chromium (pure BiFeO_3_), (e) 12.5 mol % of chromium substituting iron in the BiFeO_3_ perovskite film and (f) 25 mol % of chromium substituting iron in the BiFeO_3_ perovskite film. (g) Optical bandgap, *E*_g_, of the BiFe_1–*x*_Cr_*x*_O_3_ films as a function of the chromium content (mol % Cr) for the films on Pt/Si and on SrTiO_3_. Adapted with permission from ref (46). Copyright 2022, Published by Elsevier Ltd.

Replicating the PLD processing conditions necessary for stabilizing BiFe_1–*x*_Cr_*x*_O_3_ films through solution deposition is not feasible. However, based on the unique behavior of CSD thin films during annealing, our group has developed a new solution-processing strategy to fabricate and stabilize HP metastable perovskite thin films.^46^ This method applies external compressive stress during crystallization to the deposited layer on a compressive SrTiO_3_ substrate. This additional stress, combined with that imposed by the substrate, enables the formation and stabilization of the HP metastable BiFe_1–*x*_Cr_*x*_O_3_ phase. The CSD processing method is illustrated schematically in Figure 10a–c. Figure 10d–f shows the XRD patterns of BiFe_1–*x*_Cr_*x*_O_3_ films with varying chromium content deposited on SrTiO_3_ substrates, with and without the additional processing stress applied via the stress-mediated CSD method. Films with high chromium content could not be fabricated by conventional CSD methods. The stress-mediated CSD process was necessary to achieve chromium incorporation up to a maximum of 25 mol % in BiFe_0.75_Cr_0.25_O_3_ films. Figure 10g illustrates the bandgap reduction with increasing chromium content, achieving a minimum bandgap of ∼2.57 eV within the visible range, making these films highly promising for photovoltaic applications.

**Figure 11:**
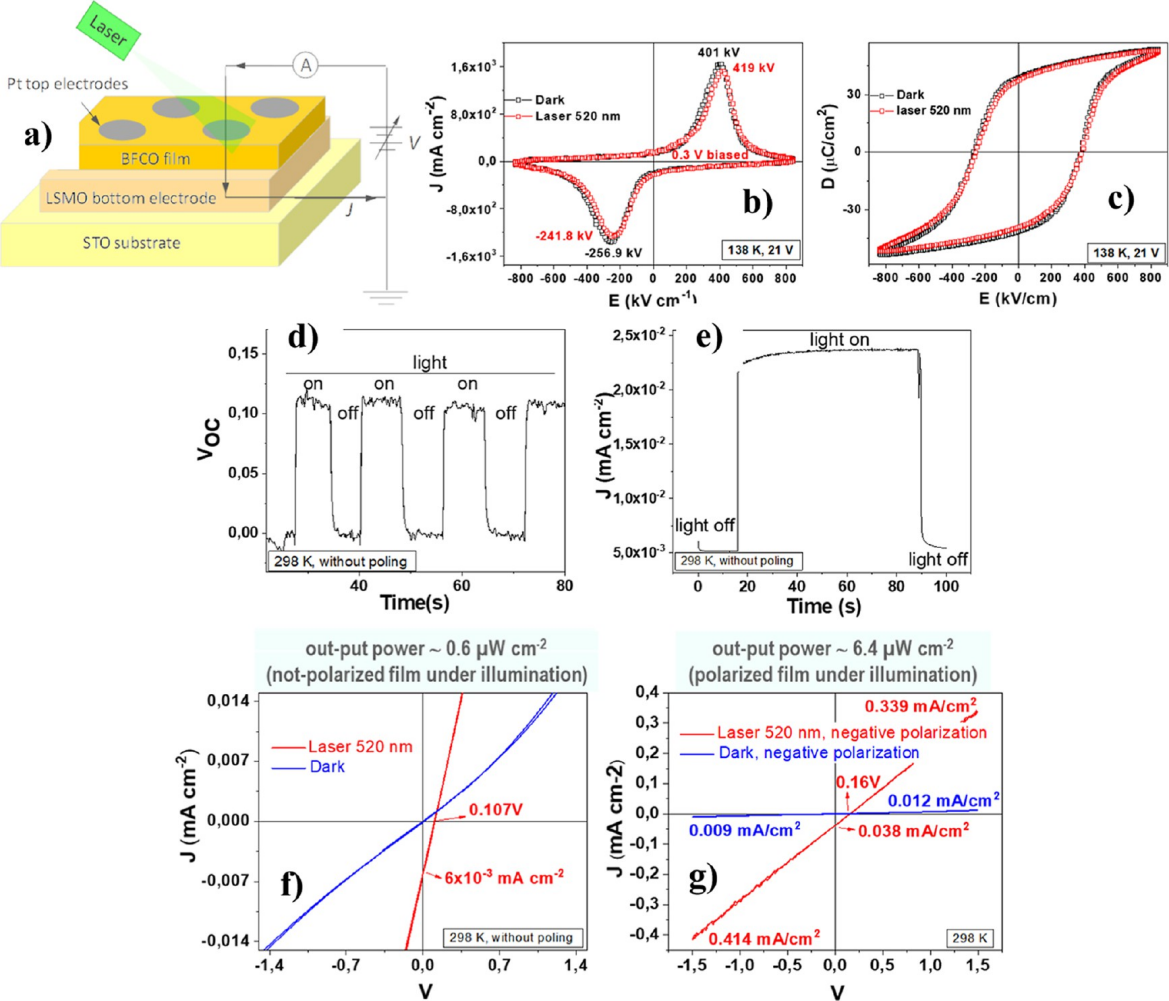
Ferroelectric (FE) and photovoltaic (PV) characterization of the ∼200 nm thick HP 12.5-BFCO double-perovskite film deposited on a La_0.7_Sr_0.3_MnO_3_ coated (100)SrTiO_3_ (LSMO/STO) substrate. (a) Scheme of the electrical configuration of the device, using planar capacitors for the ferroelectric polarization of the films and for the measurement of the photoresponse of the films with/without illumination. (b) Experimental charge current hysteresis loops, *J*–*E*, measured at 138 K in the film without and with illumination. (c) Polarization hysteresis loops, *P*–*E*, calculated by the integration of the former *J*–*E* loops. (d) Open circuit potential (*V*_OC_) measured at room temperature (RT) in the not-polarized film as a function of time, with and without illumination. (e) Time dependence of short-circuited photocurrent density measured at room temperature (RT) in the not-polarized film and under illumination. (f) Variation of current density with voltage, *J*–*V* curves, obtained at RT in the not-polarized film, and under darkness and under illumination. (g) Variation of current density with voltage, *J*–*V* curves, obtained at RT in the polarized film, and under darkness and under illumination. Adapted with permission from ref (46). Copyright 2022, Published by Elsevier Ltd.

The ferroelectric and photovoltaic responses of these BiFe_1–*x*_Cr_*x*_O_3_ double perovskite films were investigated in devices fabricated with ∼200 nm thick films as the sole light absorber. These films were deposited on La_0.7_Sr_0.3_MnO_3_-coated (100) SrTiO_3_ (LSMO/STO) substrates and equipped with very thin Pt top electrodes (Figure 11a). Figure 11b,c display the current vs electric field (*J*–*E*) curve and the ferroelectric hysteresis (*P*–*E*) loops measured at low temperature for a device containing a HP BiFeO_3_ film with 12.5 mol % chromium substitution (BiFe_0.875_Cr_0.125_O_3_). High values of remnant polarization (*P*_R_ ∼ 40 μC cm^–2^) are achieved, only slightly lower than those in pure BiFeO_3_ films. This decrease in *P*_R_ supports the incorporation of chromium into the HP metastable double perovskite films, as the reduction in the perovskite cell’s asymmetry with increased chromium content is associated with a decrease in ferroelectric response.

The unpolarized device exhibits a photovoltaic response at room temperature, as shown in Figure 11d–g. A short circuit current density (*J*_SC_) of approximately 6 × 10^–3^ mA cm^–2^ and an open circuit voltage (*V*_OC_) of ∼0.10 V are obtained (Figure 11f). For comparison, the *V*_OC_ × *J*_SC_ product can indirectly indicate the device’s output power, yielding an output power of ∼0.6 μW cm^–2^ for the unpolarized film under illumination. Following polarization, the device displays increased *J*_SC_ and *V*_OC_ values of ∼40 × 10^–3^ mA cm^–2^ and ∼0.16 V, respectively (Figure 11g), resulting in an output power of ∼6.4 μW cm^–2^, an order of magnitude higher than that of the unpolarized device. This increased output power is attributed to the enhanced *J*_SC_, mainly due to reduced charge carrier recombination in the polarized film. Here, the ferroelectric domains function as internal junctions, facilitating the separation of photoexcited electron–hole pairs, thus demonstrating the coupling between ferroelectric and photovoltaic properties.

These findings underscore the potential of BiFeO_3_-based perovskite films as effective light harvesters and, given their multiferroic nature, for integration into self-powered multifunctional systems.

### 2.2 Solution-Deposited BiFeO_3_–Based Perovskite Films on Flexible Substrates

Previous examples of solution-derived BiFeO_3_ films deposited on rigid single-crystal substrates demonstrate the potential of these films for use in various technologies in the fields of digitalization, energy, and environmental applications.

However, in today’s world of the so-called internet of things (IoT), sustainable, inexpensive, lightweight, wireless, autonomous, and flexible devices are becoming increasingly important, as silicon-based technology is expected to be cost-prohibitive for the large-scale consumer demand anticipated in the coming years.

In this context, unconventional applications are expected for flexible devices based on BiFeO_3_-based perovskite films, due to their diverse properties.

#### 2.2.1 Metal Foils Substrates

Particularly for metal foil substrates, traditional ferroelectric materials, primarily Pb(Zr,Ti)O_3_ (PZT), have been deposited on magnetic metal foils such as nickel or metglas to directly create multiferroic laminate composites.^47,48^ The advantage of these ferroelectrics on magnetic foils is their ability to produce magnetoelectricity, a property derived from the piezoelectricity and magnetostriction of the ferroic components (i.e., the ferroelectric film and magnetic substrate) through elastic coupling.^47^ However, BiFeO_3_-based perovskite compounds have been scarcely reported for these applications, despite offering undeniable advantages. A significant benefit over other ferroelectrics, such as PZT, is that BiFeO_3_ exhibits lower ferroelectric/ferroelastic domain wall activity and a higher Curie temperature, *T*_C_, (∼300 °C above that of PZT). Consequently, BiFeO_3_-based materials maintain more stable functional properties upon size reduction. This stability persists even when the microstructure is refined across the submicron range down to near the nanoscale, as is the case with thin-film materials.^49^ Additionally, this characteristic enables lower processing temperatures, minimizing metal oxidation during thermal processing of the film while retaining a suitable piezoelectric response in the fine-grained BiFeO_3_-based perovskite films.

**Figure 12:**
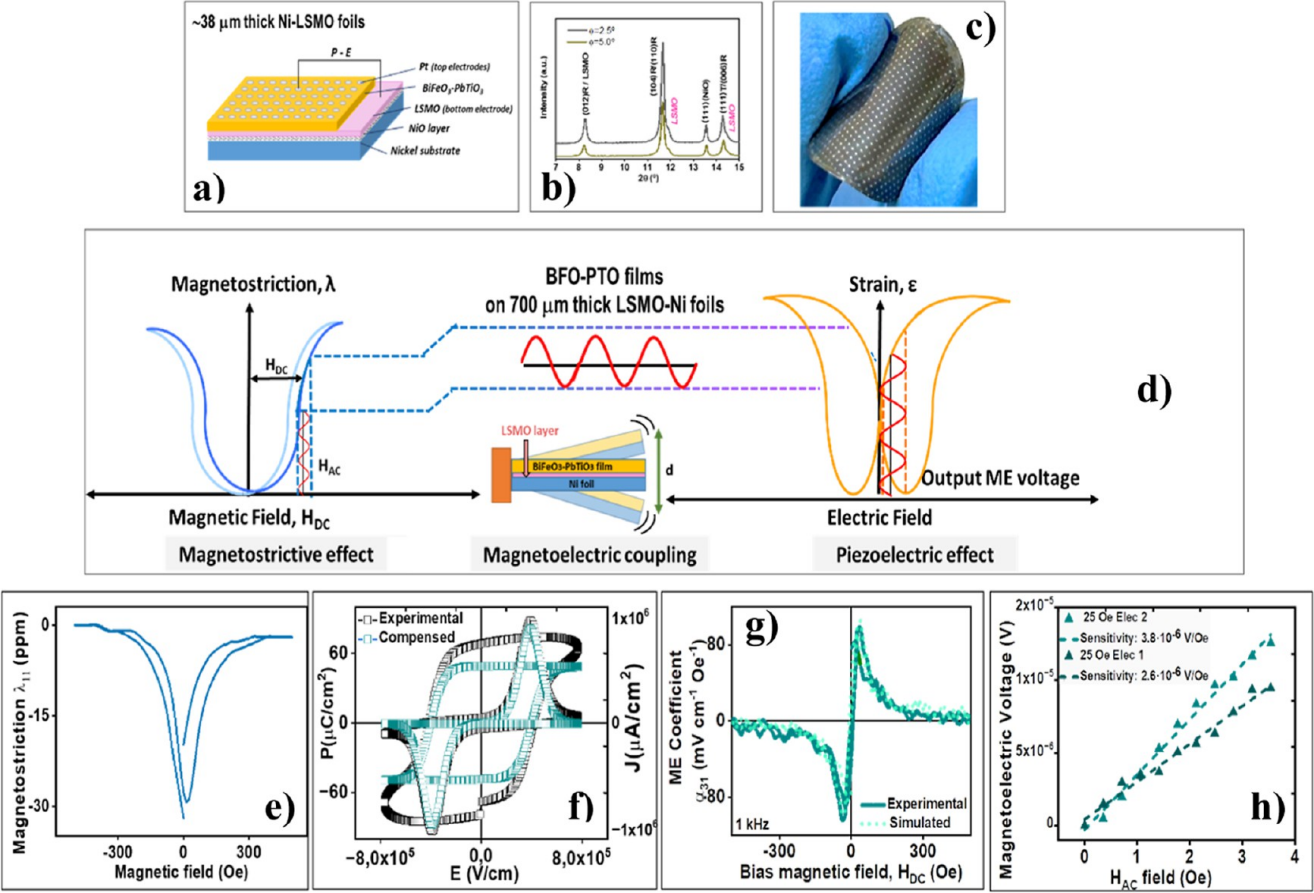
(a) Magnetoelectric device comprising a solution-deposited ferro-piezoelectric BiFeO_3_–PbTiO_3_ film on a La_0.7_Sr_0.3_MnO_3_ (LSMO) buffered ∼38 μm thick Ni foil. (b) Grazing-incidence X-ray ynchrotron iffraction (XRD) patterns of the 0.65BiFeO_3_-0.35PbTiO_3_ films on LSMO buffered Ni substrates. (c) Photograph of the flexible magnetoelectric device comprising a solution-deposited ferro-piezoelectric 0.65BiFeO_3_-0.35PbTiO_3_ film on a ∼38 μm thick LSMO buffered Ni foil. (d) Scheme of the magnetoelectric (ME) coupling process. The magnetostrictive material magnetizes and deforms in the presence of an external magnetic field H, inducing mechanical strain that is transferred across the interface to the piezoelectric layer. As a result, the ferro-piezoelectric material generates an output ME voltage. (e) magnetostriction experimentally measured in a ∼38 μm thick Ni foil. (f) Current and polarization hysteresis responses as a function of the applied electric field (*J*–*E* curves and *P*–*E* loops) for the flexible 0.65BiFeO_3_-0.35PbTiO_3_ films on a LSMO buffered Ni foil. (g) Experimental and simulated transverse magnetoelectric (ME) coefficient measured for a 0.65BiFeO_3_–0.35PbTiO_3_ film on a LSMO buffered Ni foil. (h) Induced ME voltage vs *H*_AC_ frequency at a constant *H*_DC_ of 25 Oe measured in two electrically poled electrodes, showing the linearity of the response for small *H*_AC_ and the sensitivity of the device calculated from the slopes of the fitted lines.

Our group has developed high-sensitivity magnetoelectric composites based on a thin-film geometry, as shown in Figure 12a.^50^ To achieve an optimized functional response, we have considered various aspects of this magnetoelectric device. First, a BiFeO_3_-based solid solution–primarily composed of the BiFeO_3_ multiferroic perovskite and 35 mol % of PbTiO_3_ ferroelectric perovskite (at a rhombohedral-tetragonal morphotropic phase boundary, MPB, known for strong piezoelectric activity)—was selected due to the reported benefits this compound offers at the nanoscale. Second, nickel (Ni) foils were chosen as the magnetic substrate, as nickel is an inexpensive material with significant magnetostriction. The relatively low solution processing temperatures (500 °C) and the rapid thermal processing (RTP) treatments used to fabricate these BiFeO_3_-based perovskite films have allowed us to kinetically limit nickel oxidation. Additionally, the solution deposition of a conductive buffer layer of La_0.7_Sr_0.3_MnO_3_ (LSMO) perovskite significantly improved the ferro-piezoelectric/Ni interface. This fabrication strategy for the magnetoelectric device (Figure 12a) enables the crystallization of single-phase ferroelectric films without appreciable interdiffusion across the film–substrate interface, which would otherwise lead to the formation of secondary phases detrimental to the films’ functional response. Accordingly, the X-ray synchrotron radiation diffraction (XRD) patterns collected with variable incidence angles (2.5 and 5°) for films directly deposited on LSMO-buffered Ni substrates (Figure 12b) indicate the formation of a single perovskite phase, predominantly with a randomly oriented rhombohedral crystal structure, without detection of secondary phases. Figure 12c shows a photograph of the fabricated magnetoelectric device, comprising the BiFeO_3_–PbTiO_3_ ferro-piezoelectric film on a ∼38 μm thick Ni foil.

Figure 12d schematically illustrates the magnetoelectric (ME) coupling process in these flexible materials. When an external magnetic field (H) is applied to the device, the magnetostrictive flexible Ni substrate magnetizes and deforms, inducing mechanical strain in the ferro-piezoelectric layer. Consequently, the ferro-piezoelectric material generates an output ME voltage, enabling the device to function as both a magnetic field sensor and an efficient energy harvester.

Magnetostriction was experimentally measured in the Ni foil (Figure 12e). Additionally, ferroelectric hysteresis loops (*P*–*E* loops and *J*–*E* curves) with relatively low coercive fields were obtained at room temperature in capacitors made from the flexible material (Figure 12f), indicating low leakage currents in the film at room temperature. This suggests that these flexible materials exhibit robust ferroelectric behavior under ambient conditions, which is essential for energy harvesting applications. Transverse ME coefficients, α_31_, were both experimentally measured and theoretically calculated, considering the properties of the device’s magnetic and ferro-piezoelectric components. Experimental α_31_ values of approximately 100 mV cm^–1^ Oe^–1^ were attained, which are below those expected from the analytical solution for a two-layer system and bulk piezoelectric coefficients, assuming ideal elastic coupling.^50^ This indicates a nonideal coupling and reduced film piezoelectric coefficients, obtaining a good agreement for an interfacial coupling *k* value of 0.3, when a piezoelectric *d*_31_ coefficient of ∼40 pC N^–1^ is used (Figure 12g). As shown in Figure 12h, the induced ME voltage measured across two electrically poled electrodes displays a clear linear dependence on the amplitude of the AC magnetic field (*H*_AC_). This linear relationship is essential for reliable sensing performance, ensuring a consistent and predictable response. The slope of this linear response, between 2.6 and 3.8 μV Oe^–1^, represents the sensitivity of a hypothetical sensor.

Thus, these flexible BiFeO_3_-based magnetoelectric materials, fabricated via solution deposition (CSD) using cost-effective, low thermal budget procedures, demonstrate excellent magnetoelectric transduction capabilities, underscoring the potential of these solution-derived flexible structures for magnetic field sensing and harvesting applications.

#### 2.2.2 Flexible Plastic Substrates

The advancement of Flexible Electronics has been driven by key demands in the current electronics industry, particularly the need for cost-efficient, portable, high-tech devices. In these flexible devices, the active layer is typically supported on affordable, flexible plastics with degradation temperatures ≤400 °C (e.g., polyimides (PI), which have the highest glass transition temperatures among plastics).^51^ Amorphous metal oxide semiconductors are the most widely used active layers in flexible electronics due to their relatively low processing temperatures.^52^ However, crystalline metal oxides, such as BiFeO_3_-based perovskites, require processing temperatures that exceed the thermal tolerance of plastic substrates.^53^ As a result, Flexible Electronics has driven the development of low-temperature fabrication processes that enable the deposition of high-performance thin layers on temperature-sensitive plastic substrates. Furthermore, flexible electronics also demands film deposition techniques that scale effectively to accommodate the dimensions of flexible devices. Solution-based methods are particularly well-suited to meet these requirements.^22,25−28^

##### 2.2.2.1 Low-Temperature Solution Deposition Film Processing

**Figure 13:**
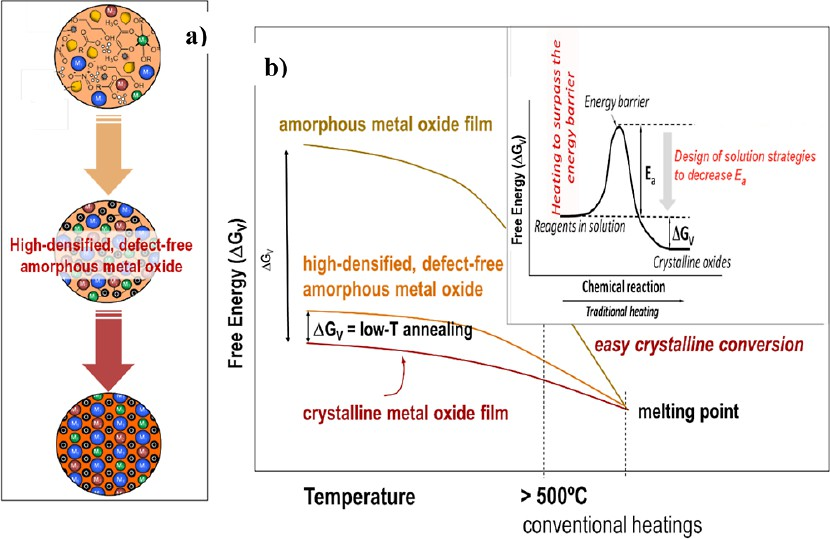
(a) Scheme showing the three major steps to obtain crystalline thin films from solutions, starting from a solution layer with a large content of organics, porosity and carbonaceous residues, which is converted into a high-density, defect-free amorphous metal oxide film and then into a crystalline film with an ordered array of the metal and oxygen atoms. (b) Diagram of the free energies of a solution derived amorphous film and the crystalline perovskite phase (Δ*G*_V_ = driving force for oxide crystallization). Inset shows the energy activation barrier, Ea, that should be surpassed for the amorphous to crystalline conversion of the metal oxide. Adapted with permission from ref (28). Copyright 2023, Springer Nature.

All the low-temperature solution strategies reported in the literature for the fabrication of crystalline (as well as high-density, defect-free amorphous) metal oxide films should be directed toward the prompt implementation of the processing pathway described in Figure 13.^25−28^ This is related to the fact that the as-deposited coatings derived from solutions are always amorphous materials containing a large amount of organic compounds. The only way to accelerate the nucleation and growth of the crystal phase is through the rapid elimination of these organics, facilitating the formation of an interconnected metal–oxygen (−M–O–M−) network, which serves as the building block of the crystalline metal oxide material (Figure 13a). If the solution deposition strategy employed is capable of minimizing the content of defects, such as pores, solid particles, occluded solvent or organics, and thus obtaining a highly densified (close-packed −M–O–M– network) amorphous metal oxide layer, then the activation energy (*E*_a_) barrier that needs to be surpassed for film crystallization is reduced. Consequently, a lower amount of thermal energy would be required to achieve the ordered atomic arrangement in the crystalline film (Figure 13b).

In our group, we have developed various low-temperature solution-deposition strategies for fabricating BiFeO_3_-based perovskite films on polyimide, resulting in flexible materials with promising potential for various applications. These solution methods have enabled the direct preparation of functional BiFeO_3_ thin films on plastic substrates with thicknesses below 500 nm using CSD at a lower temperature range of 300–350 °C, approximately 200 °C below the temperatures typically used for depositing BiFeO_3_ perovskite films on rigid single-crystal substrates via conventional CSD methods. Some of these strategies are described below.

**Figure 14:**
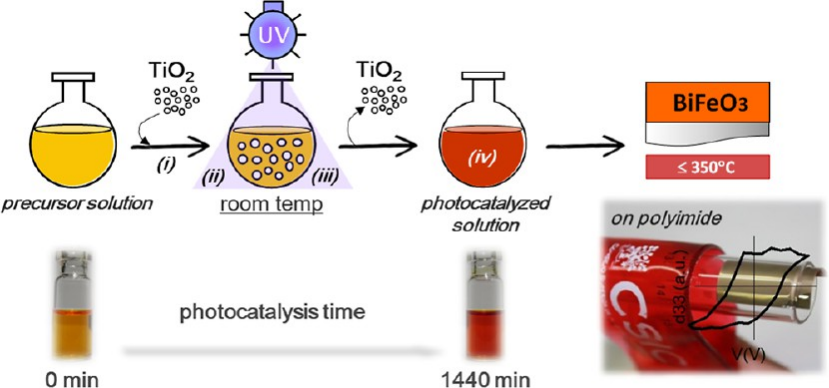
Heterogeneous photocatalysis of precursor solutions to create low-temperature liquid precursors, enabling the direct solution deposition of ferroelectric BiFeO_3_ perovskite thin films on flexible polyimide substrates. Adapted with permission from ref (54). Copyright 2015 Wiley-VCH Verlag GmbH & Co. KGaA.

Heterogeneous photocatalysis of precursor solutions is an approach that directly modifies the chemistry of the synthesized BiFeO_3_ precursor solution to reduce its organic content, thereby producing a low-temperature liquid precursor.^54^ In this approach, photocatalytic TiO_2_ particles are added to the precursor solution, and the resulting suspension is illuminated with UV light. During irradiation, the bismuth and iron reagents near the TiO_2_ particle surfaces undergo photocatalytic reactions, induced by the TiO_2_ particles upon light absorption. This process chemically breaks down most of the organic components in the solution, promoting their decomposition directly within the solution. After this step, the TiO_2_ particles are separated from the solution by centrifugation, resulting in a photocatalyzed liquid precursor with reduced organic content. This precursor can then be directly deposited on flexible polyimide substrates to produce BiFeO_3_-perovskite films at temperatures below the degradation threshold of the substrate (Figure 14).

**Figure 15:**
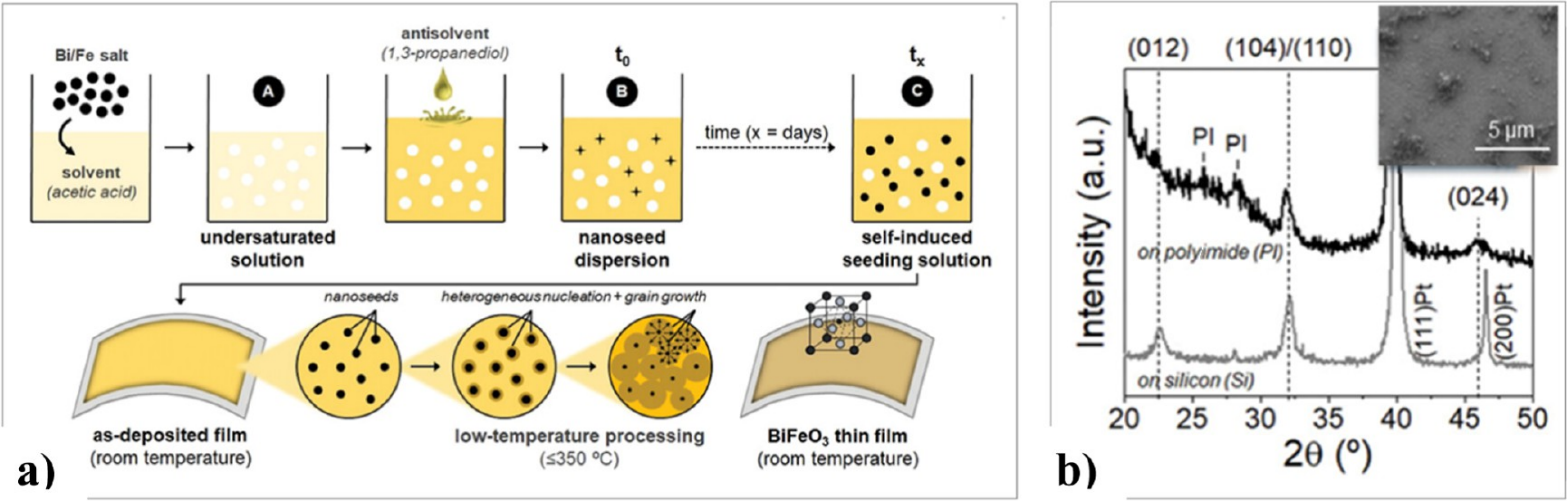
(a) Schematic of the solvent-engineering method for low-temperature processing of flexible BiFeO_3_ thin films on polyimide (PI). The process begins with the dissolution of metal salts to obtain an undersaturated solution (A). This is followed by the initial formation of a nanoseed dispersion upon adding an antisolvent (B), leading to the creation of a self-induced seeding solution (C), which is subsequently deposited onto a substrate. (b) X-ray diffraction (XRD) pattern of the BiFeO_3_ thin film processed at 350 °C on flexible polyimide and rigid silicon. The inset shows a scanning electron microscopy (SEM) image of the surface of single-layer films deposited from the self-induced seeding solution and dried at 150 °C. Adapted with permission from ref (56). Copyright 2020 Wiley-VCH Verlag GmbH & Co. KGaA.

A well-known approach for reducing the crystallization temperature of metal oxide films is the addition of crystalline nanoparticles (nanoseeds) to the precursor solution.^55^ Once these solutions are deposited onto the substrate, the nanoseeds act as nucleation centers, promoting the formation and growth of the crystalline metal oxide film. The main disadvantage of this low-temperature solution method is that the crystalline particles must be fabricated outside the solution at high temperatures. We have developed a novel self-induced solution seeding approach for forming nanoparticles directly within BiFeO_3_ perovskite precursor solutions.^56^ This strategy involves the controlled addition of an antisolvent, 1,3-propanediol, to an acetic acid solution containing Bi(III) and Fe(III) salts, resulting in the in situ formation of nanoparticles (seeds) by supersaturation within the precursor solution. Although we have not yet determined whether these nanoparticles are crystalline, the experimental results show that these nanoseeds accelerate the crystallization of the perovskite phase in the solution-deposited layer. This enables the direct growth of BiFeO_3_ perovskite films on polymeric substrates at low temperatures (Figure 15).

**Figure 16:**
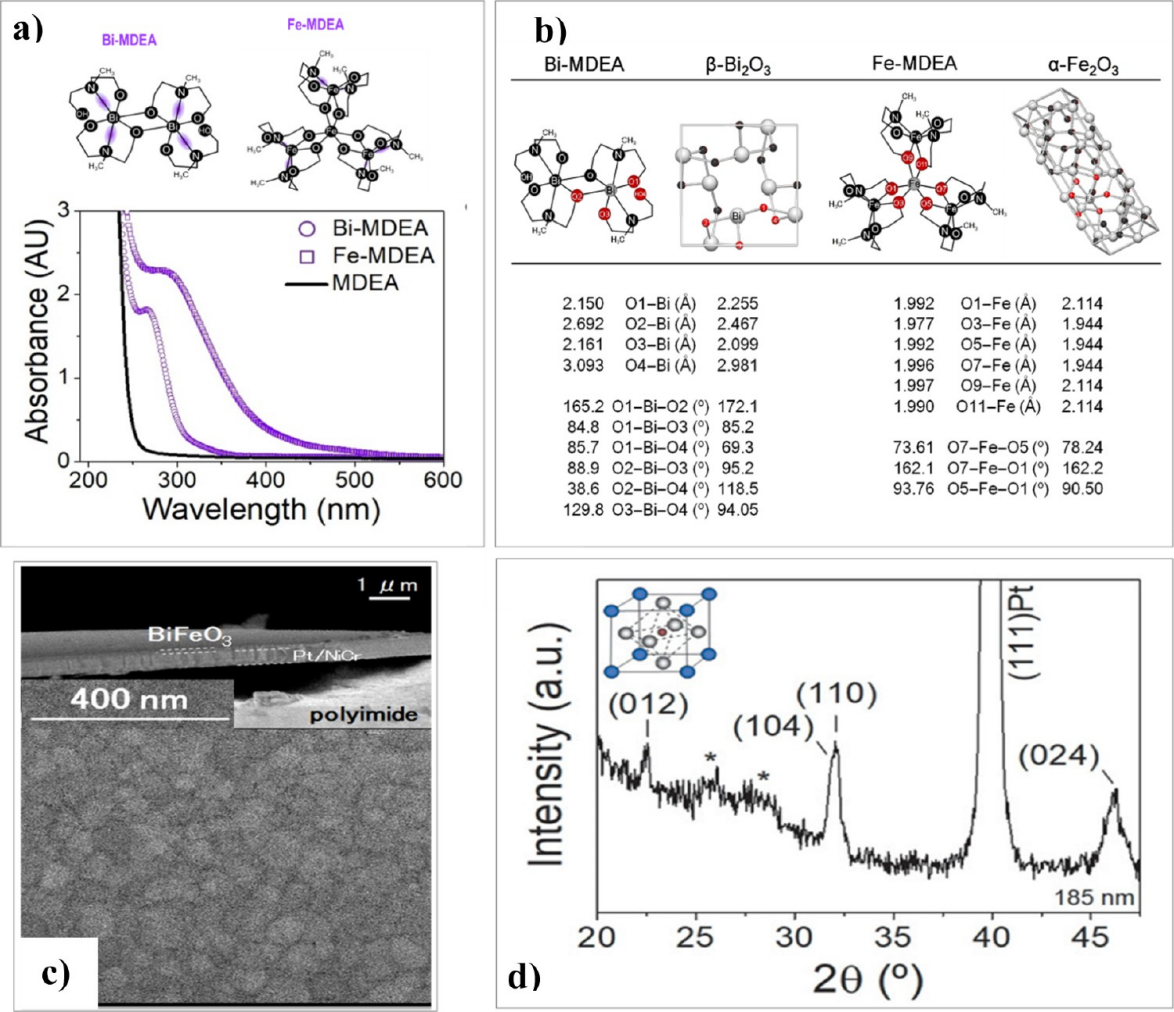
(a) Vis-UV absorption spectra of the metal precursors, showing in the molecular structures of the metal complexes the bonds (in violet) responsible for their high UV absorptivity. (b) Molecular structures of the Bi-MDEA and Fe-MDEA precursors alongside the crystalline structures of their corresponding metal oxides, highlighting the similarity between bond lengths and angles in the molecular and crystalline structures. (c) Cross-sectional and surface scanning electron microscopy (SEM) images of the BiFeO_3_ perovskite film on flexible polyimide. (d) X-ray diffraction (XRD) pattern of the BiFeO_3_ film deposited at low temperature on a Pt-coated flexible polyimide substrate. Asterisks (*) indicate reflections from the polyimide substrate. Adapted with permission from ref (58). Copyright 2020 Wiley-VCH Verlag GmbH & Co. KGaA.

Our group has worked extensively on developing strategies based on PhotoChemical Solution Deposition (PCSD) for thin films, which involves using UV irradiation to assist in film fabrication at low temperatures.^57^ These strategies focus on synthesizing photosensitive precursor solutions that must contain photosensitive species. Charge-transfer metal coordination complexes are ideal for this purpose due to their high absorption in the UV range. We have synthesized metal complexes of bismuth and iron with *N*-methyldiethanolamine (Bi-MDEA and Fe-MDEA) in solution, forming coordination complexes characterized by metal–nitrogen bonds responsible for their high UV absorptivity (Figure 16a). Figure 16b shows the structure of these molecular complexes and their corresponding crystalline oxides, with selected bond lengths and angles highlighted in red for comparison. Although minor deviations are observed in some bond distances and angles, the resemblance between the respective molecular and crystalline structures is relatively high for both systems (Bi-MDEA and Bi_2_O_3_, and Fe-MDEA and Fe_2_O_3_). Thus, the optimal atomic arrangement in these Bi-MDEA and Fe-MDEA precursors, with a homogeneous distribution of molecular clusters containing Bi(III), Fe(III), and oxygen, facilitates the mass transport necessary for crystalline oxide formation through atomic diffusion under a kinetic regime. Combined with UV irradiation to expedite organic removal and densification, this solution method enables the fabrication of BiFeO_3_ films at temperatures compatible with flexible plastic substrates.^58^ Thus, Figure 16c shows the cross-section and surface image of the BiFeO_3_ films directly deposited on polyimide. Additionally, Figure 16d shows the X-ray diffraction pattern of these flexible BiFeO_3_ perovskite films.

The atmosphere in which the solution-derived film is processed can significantly impact the low-temperature fabrication of BiFeO_3_ perovskite films. Generating specific processing atmospheres is especially relevant in photochemical solution deposition (PCSD), as UV light can not only trigger chemical reactions in the irradiated film but also produce reactive species in the processing atmosphere. The irradiation of gas molecules leads to their photolysis, resulting in the formation of reactive free radicals. These radicals initiate secondary reactions, such as attacking gas molecules to form new radicals and molecular species in the atmosphere. These reactive species can accelerate the formation of the intermediate amorphous metal oxide film and promote earlier crystallization. We refer to this approach as film crystallization induced by photochemically generated reactive radicals.^59^

**Figure 17:**
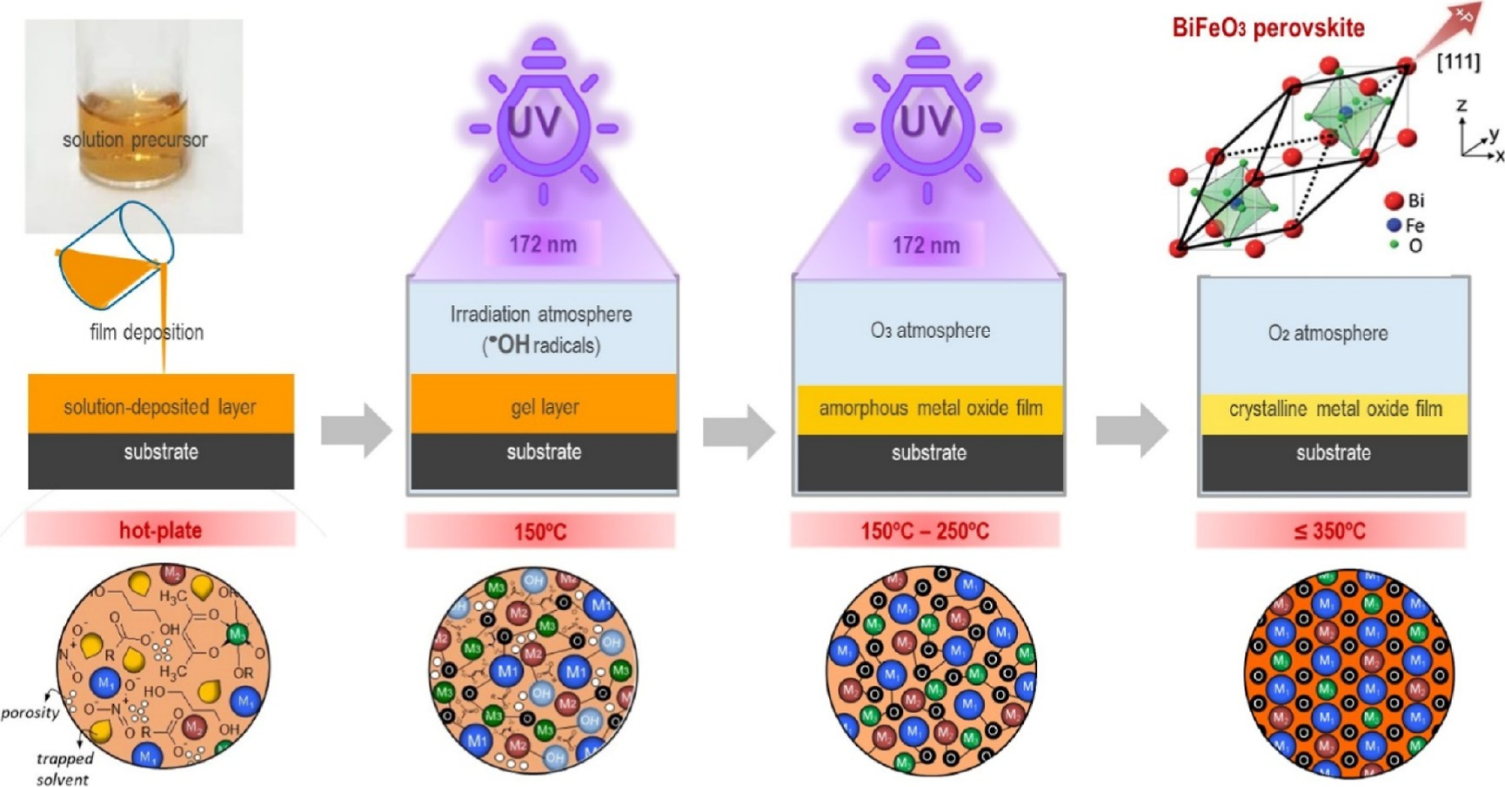
Hydroxyl (HO^•^) radical method for accelerating the formation and crystallization of solution-deposited BiFeO_3_ thin films, illustrating the different steps of the process: (1) solution deposition of the film onto the substrate, (2) UV irradiation of the deposited layer at λ = 172 nm in a HO^•^ atmosphere, (3) further UV irradiation of the film in an O_3_ atmosphere, and (4) crystallization of the perovskite film at temperatures below 350 °C. The drawings at the bottom depict the characteristics of the films obtained after each step of the process.^59^

Oxygen (O_2_) atmospheres are commonly used during UV irradiation of solution-deposited metal oxide thin films, as the photolysis of O_2_ produces oxygen radicals (O^•^), which react with molecular O_2_ to form ozone (O_3_), a strong oxidizing agent that enhances decomposition of organic compounds in the film. Figure 17 shows a specific case where O_2_ atmospheres enriched with water vapor (H_2_O) produce HO^•^ radicals. These highly reactive HO^•^ species rapidly attack the chemical compounds in the solution-derived film, enhancing the condensation of the metal–oxygen network and promoting the formation of a highly densified, defect-free amorphous metal oxide film. This film can then crystallize with minimal energy input, such as low-temperature thermal treatments. Consequently, this solution-based strategy enables the low-temperature crystallization of flexible BiFeO_3_ perovskite films.

##### 2.2.2.2 Properties of Flexible BiFeO_3_ Thin Films

Next, the applicability of these low-temperature solution deposition methods for the direct fabrication of BiFeO_3_-perovskite films onto flexible plastic substrates is demonstrated. Based on their properties, these flexible materials show potential for emerging technologies when capable of operating in real devices. However, it should be noted that preparing functional films on flexible substrates, even at low temperatures, is much more challenging than on rigid, single-crystal substrates with atomically flat surfaces. On the one hand, the mechanical incompatibility between the brittle metal oxide (BiFeO_3_ film) and the flexible plastic substrate can impact the physical properties of the oxide layer at macroscopic, micro, and nanoscale levels. Typically, substrate conditioning treatments prior to film deposition are recommended.^55^ These treatments aim to increase the smoothness of the plastic substrate surface, reduce its shrinkage during drying or pyrolysis, and prevent peeling or cracking of the film during crystallization.

Therefore, it should be taken into account that the functional properties of low-temperature solution-processed crystalline oxide films on flexible plastic substrates will be always lower than those of counterpart films processed at high temperatures on single-crystal substrates. This is partly due to the very different mechanical properties of the inorganic crystalline film and the flexible organic substrate, which make accommodation at the film–substrate interface difficult and hinder the full utilization of the film’s properties. Additionally, in the case of ferroelectric films like BiFeO_3_, the nanometer-sized crystalline grains resulting from low-temperature processing, along with the possible presence of small amounts of amorphous phases, can impede connectivity among ferroelectric grains, thus hindering nucleation and growth of ferroelectric domains, and reducing the ferroelectric response.

**Figure 18:**
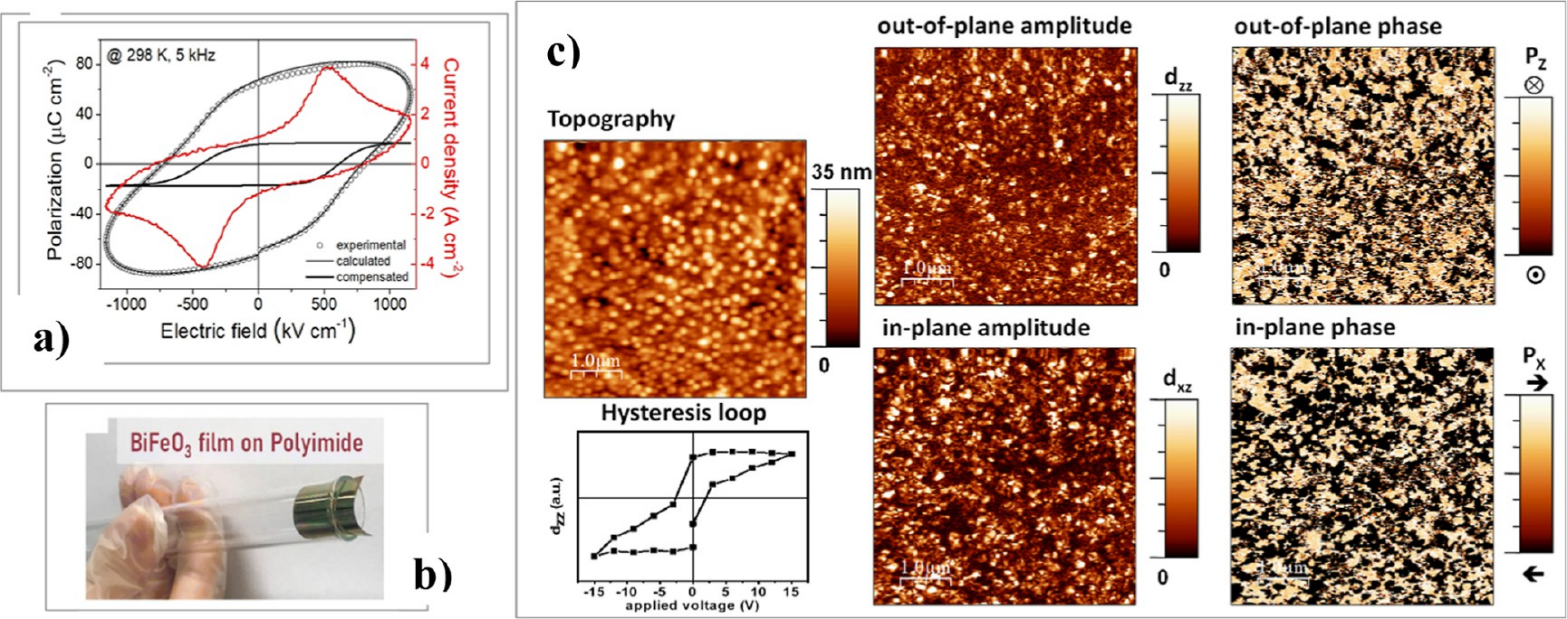
a) Ferroelectric hysteresis (*P*–*E*) loop and current vs electric field (*J*–*E*) measured in the flexible BiFeO_3_ film on polyimide. The compensated *P*–*E* loop is also shown after eliminating nonswitching contributions (capacitance, resistance, and nonlinear conduction). (b) Photograph of a BiFeO_3_ perovskite film directly deposited by a low-temperature solution method on a Pt-coated flexible polyimide substrate. (c) Topography, local piezoelectric hysteresis loop, out-of-plane and in-plane amplitude images, and out-of-plane and in-plane phase images of the flexible BiFeO_3_ thin films on polyimide. Adapted with permission from ref (58). Copyright 2023, The Royal Society of Chemistry.

Effectively, as shown in Figure 18a, the ferroelectric hysteresis loops (*P*–*E* loops and *J*–*E* curves) of these flexible films (Figure 18b) exhibit significant nonswitching contributions (capacitance, resistance, and nonlinear conduction). After compensating for these effects using a simulation model,^56^ remnant polarization values of *P*_R_ ∼11 μC cm^–2^ and a coercive field of *E*_C_ ∼530 kV cm^–1^ were obtained. These *P*_R_ values are lower than those achieved in BiFeO_3_ thin films prepared at conventional temperatures on rigid silicon substrates (see previous sections). However, they compare favorably with most ferroelectric thin films processed at low temperatures (mainly organic ferroelectrics) and meet the requirements for fabricating real ***ferroelectric devices***.^60^

As expected for these low-temperature solution-processed films, the low-magnification topographic and amplitude and phase piezoelectric images obtained by piezoresponse force microscopy (PFM) (Figure 18c) show that these films consist of equiaxed nanometric grains with a piezoelectric response homogeneously distributed over the surface. The piezoelectricity of these films is further confirmed by the hysteresis loop depicted in Figure 18c.

**Figure 19:**
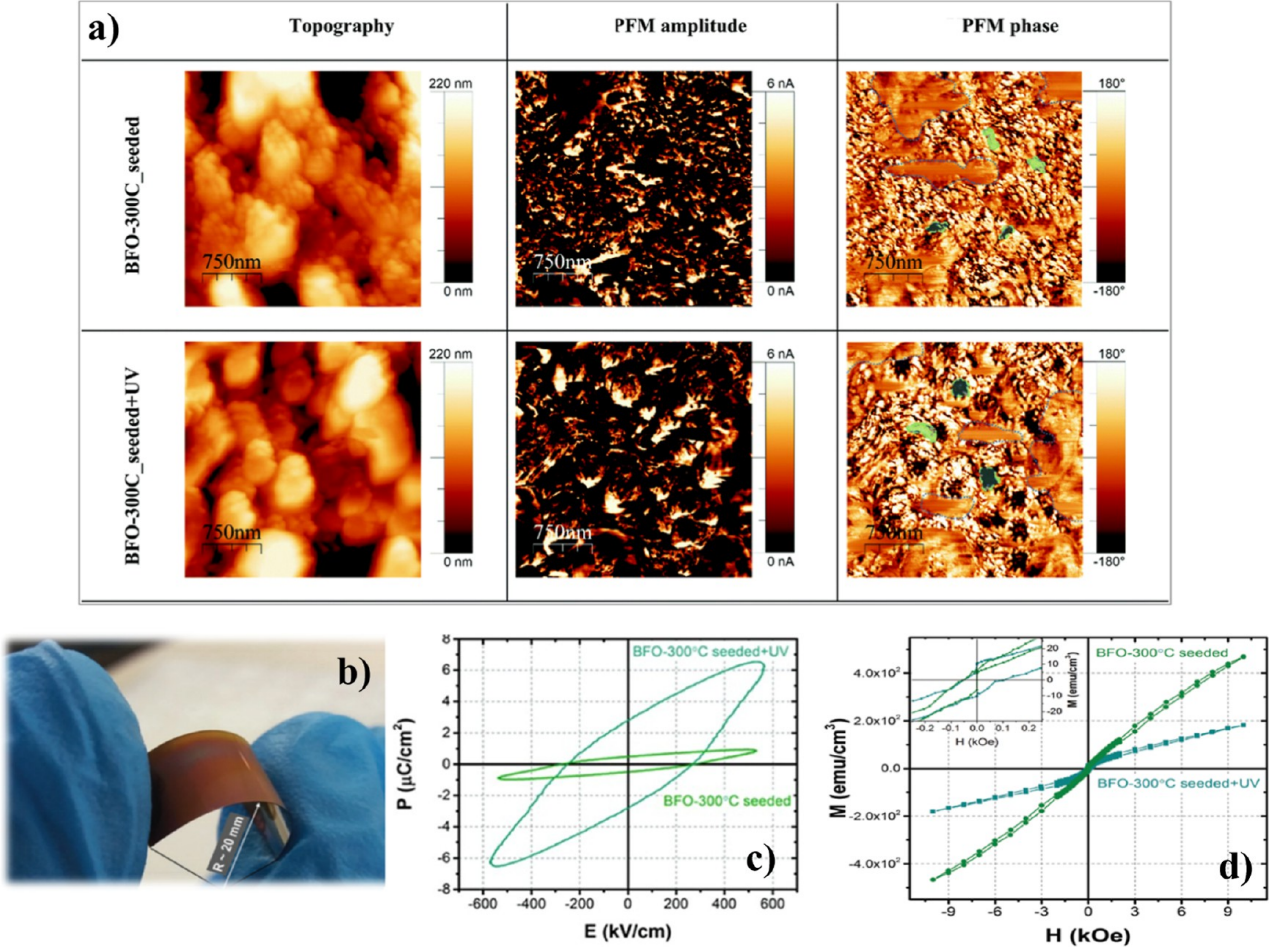
(a) Topographic and piezoresponse force microscopy (PFM) amplitude and phase images of BiFeO_3_ thin films on flexible polyimide substrates, crystallized at 300 °C for 120 min, prepared with seeded and seeded + UV irradiation methods. Both films exhibit piezoelectric behavior. Areas with poor contrast likely correspond to regions of the films with incipient crystallization and domains with in-plane polarization. Some domains are highlighted in green for comparison. (b) Photograph of one of these BiFeO_3_ perovskite films directly deposited on a Pt-coated polyimide substrate. (c) Ferroelectric (*P*–*E*) loops measured at 140 K and 10 kHz for BiFeO_3_ films derived from nonseeded and seeded precursor solutions. (d) Magnetic (M-H) loops measured at 300 K up to 10 kOe for BiFeO_3_ films deposited from nonseeded and seeded precursor solutions.^61^

Piezoelectric and ferroelectric responses were also measured in flexible BiFeO_3_ films prepared with UV irradiation and low thermal annealing at 300 °C from seeded solutions, in comparison to films prepared from nonseeded solutions. When comparing these properties, an improvement in the functional response of the seeded films is evident. See Figure 19, where the topographic and piezoresponse force microscopy (PFM) amplitude and phase images of these flexible films are shown. Both films exhibit piezoelectric behavior; however, areas of ferroelectric domains, highlighted in green for comparison, appear larger in the films obtained from seeded solutions, indicating a better ferro-piezoelectric response (Figure 19a). Figure 19b shows a photograph of one of these flexible BiFeO_3_ films directly deposited on polyimide by a low-temperature solution deposition method. The improvement in the ferroelectric and ferromagnetic responses of these films (Figure 19c,d) clearly demonstrates an enhancement of these functional properties in films processed at only 300 °C, achieved by combining the seeding of precursor solutions and UV irradiation during fabrication. This enhancement is attributed to better crystallization, higher crystallinity, and a lower content of residual amorphous phases in these films.^61^

**Figure 20:**
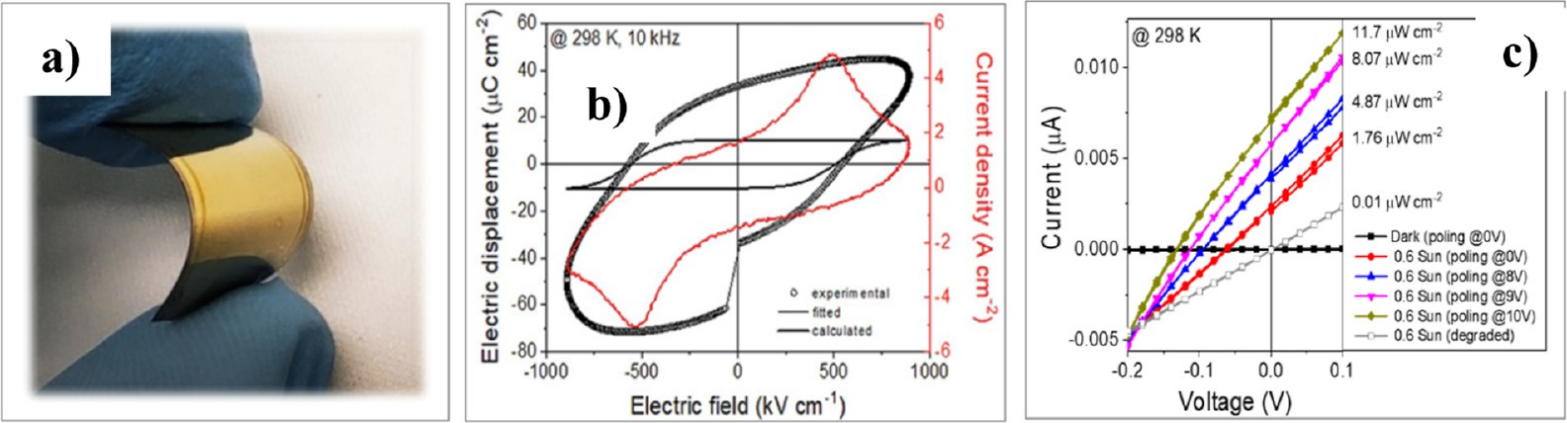
(a) Photograph of a BiFeO_3_ perovskite film directly deposited on a Pt-coated polyimide substrate from self-induced seeded solutions and crystallized at 350 °C. (b) Ferroelectric hysteresis (*P*–*E*) loop and current vs electric field (*J*–*E*) curve measured in these films at room temperature (RT) and 1 kHz. (c) Current–voltage characteristics and calculated power output values of this flexible BiFeO_3_ thin film, measured at RT under different illumination and poling conditions. Adapted with permission from ref (56). Copyright 2020 Wiley-VCH Verlag GmbH & Co. KGaA.

The potential of these BiFeO_3_ films on flexible polyimide substrates for use in ***photovoltaic devices*** is demonstrated in Figure 20. This figure shows a photograph of a flexible BiFeO_3_ film deposited from self-induced seeded solutions and its ferroelectric behavior at room temperature (RT) (Figure 20a,b). Figure 20c displays the current–voltage (*J*–*V*) characteristics of this flexible BiFeO_3_ film under different illumination and poling conditions. Without electric poling, the film exhibits a photovoltaic effect at room temperature under illumination (0.6 Sun), yielding a small photocurrent density (30 μA cm^–2^). This behavior is attributed to self-polarization typically observed in ferroelectric thin films, resulting from strain gradients generated in the perovskite lattice due to substrate clamping. Both short-circuit current density and open-circuit voltage increase with poling, reaching maximum values of 90 μA cm^–2^ and 0.13 V. From these results, a power output of 11.7 μW cm^–2^ is calculated, with an extrapolated value of ∼20 μW cm^–2^ at 1 Sun, which is an order of magnitude higher than that of the nonpoled sample.^56,58^

The built-in electric field generated by the formation of ferroelectric domains in the thin film is directly responsible for the observed photovoltaic effect, with each domain boundary acting like a classical p–n junction that separates photogenerated electrons and holes, thereby enhancing the device’s properties under illumination. These results confirm the coupling between ferroelectric polarization and the photovoltaic effect in these photoferroelectric BiFeO_3_ films. While the power output obtained for the flexible BiFeO_3_ thin film is low for energy conversion applications (e.g., in solar cells), this output is sufficient to meet the requirements for self-powered devices (micropower operation) based on energy harvesting from light.

**Figure 21:**
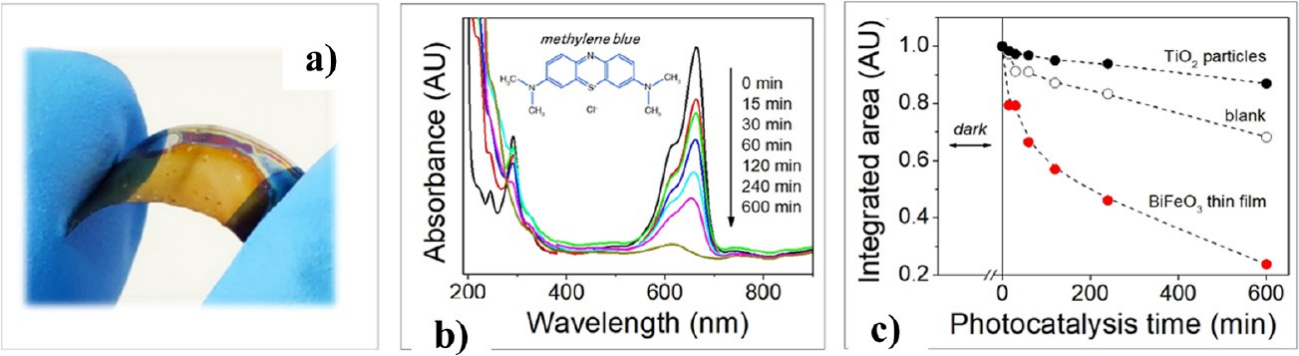
(a) Photograph of a BiFeO_3_ perovskite film directly deposited on a Pt-coated polyimide substrate at 350 °C. These films were deposited from solutions containing photosensitive molecular Bi-MDEA and Fe-MDEA complexes, which have molecular structures similar to those of their corresponding crystalline oxides, and were crystallized at 350 °C. (b) Evolution over time of the Vis-UV absorption spectra of methylene blue on a flexible BiFeO_3_ thin film under visible illumination. (c) Evolution of the relative integrated area corresponding to the 550–700 nm absorption bands of methylene blue under visible light by a flexible BiFeO_3_ thin film as a function of photocatalysis time. Adapted with permission from ref (58). Copyright 2020 Wiley-VCH Verlag GmbH & Co. KGaA.

Apart from photovoltaics, other photoinduced effects in photoferroelectrics may provide additional functionalities and coupling phenomena, offering high potential for novel and future applications in various fields. One example is the photocatalytic activity demonstrated by the flexible BiFeO_3_ films in this work.^56,58^Figure 21a shows a flexible BiFeO_3_ film that was prepared at a low temperature of 350 °C with the assistance of UV irradiation and from solutions containing Bi-MDEA and Fe-MDEA complexes. Figure 21b illustrates the degradation of the organic dye methylene blue (MB) over this flexible film through photocatalytic oxidation using visible light. After 600 min of irradiation, the degradation rate of MB (calculated from the relative integrated area of the absorption bands between 550 and 700 nm) reached 76% (Figure 21c), which was more than twice the value obtained without the sample (i.e., the blank). For comparison, the photodegradation of MB using commercial particles of the traditional TiO_2_ photocatalyst is also shown, revealing that the degradation remains practically unaltered with the TiO_2_ particles, as TiO_2_ has a higher bandgap than BiFeO_3_. This efficient photocatalytic activity of these flexible BiFeO_3_ thin films may provide significant benefits to the industrial sector in terms of cost, utilizing inexpensive, lightweight, and flexible polymer substrates.^58^ Additionally, novel applications based on flexible photocatalytic systems could emerge in construction and energy (e.g., self-cleaning windows, solar panels, or H_2_ generation) and environmental remediation (e.g., external transparent pipes for wastewater treatment), where the economic and toxicity issues typically associated with the removal of photocatalytic nanoparticles from the system would be avoided.

Therefore, the successful integration of these films with flexible polyimide through low-temperature solution deposition methods, along with the broad range of properties exhibited by these flexible BiFeO_3_ materials, opens new opportunities not only in various subfields of flexible electronics, such as autonomous wearable electronics, but also in applications related to energy and the environment (e.g., self-powered devices, sustainable remediation systems, and large-area self-cleaning devices).

## 3 Outlook, Future Prospects and Opportunities

**Figure 22:**
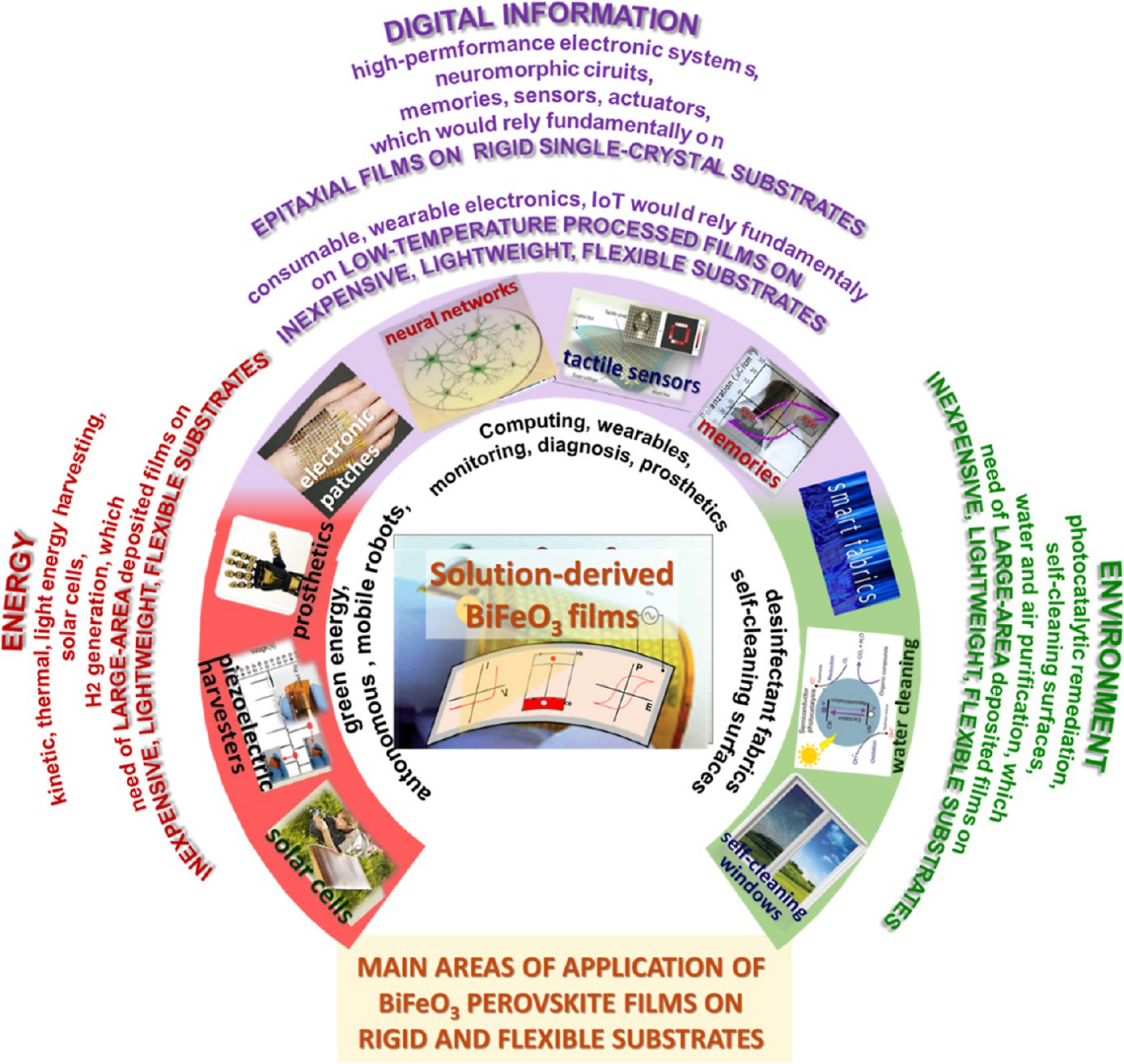
Potential applications of solution-derived BiFeO_3_-based perovskite films in emerging technologies encompass areas such as digitalization, energy, and the environment.

Figure 22 shows a snapshot of the potential of BiFeO_3_-based perovskite films in three major fields of application: Digital Information, Energy and Environment.

Among these features, the primary focus on BiFeO_3_-based perovskite thin films over the past decade has been their integration into devices for applications in the area of Digital Information. Potential devices utilizing single-crystal substrates have harnessed the multiple properties of BiFeO_3_, including ferroelectricity, (anti)ferromagnetism, piezoelectricity, and magnetoelectricity. These properties provide notable advantages for low-power, high-performance electronic devices such as memories, sensors, actuators or neuromorphic systems. This is primarily due to their large spontaneous polarization (*P*_S_ ∼100 μC/cm^2^), high Curie temperature (*T*_C_ ∼1103 K), and the coexistence of ferroelectric and magnetic properties at room temperature. Typically, high-quality epitaxial BiFeO_3_ perovskite thin and ultrathin films are required for these applications.

BiFeO_3_-based films with these characteristics can be fabricated using chemical solution deposition (CSD) on rigid single-crystal substrates. However, the high cost of these substrates (e.g., Si semiconductor substrates or SrTiO_3_) and the expensive micromechanization techniques (e.g., etching or lithography) required for achieving small size, high integration density, fast switching, and low-power consumption make them less cost-effective for these applications. Another significant drawback of BiFeO_3_ films is their high leakage currents, which hinder the application of these devices at room temperature. This challenge remains unresolved due to the difficulty in preventing bismuth loss at the conventional processing temperatures (>500 °C) required for crystallizing these films, as well as the complexity of controlling the valence fluctuation of Fe^3+^ to Fe^2+^.

Partially substituting Bi and Fe in the perovskite lattice with other cations or incorporating suitable buffer layers has been explored to enhance the electrical performance of these films, aiming to make them viable for room-temperature applications. However, these solutions often involve introducing toxic, critical and strategic elements, such as Mn(III), La(III), and Cr(III), which undermines sustainability. Although BiFeO_3_ itself is a nontoxic compound that does not contain critical elements, these additions compromise the environmental and economic viability of the material. These challenges have significantly hindered the development of BiFeO_3_ thin-film devices on rigid single-crystal substrates.

In this context, CSD presents unique opportunities that other deposition techniques cannot offer. On one hand, low-cost, high-throughput solution deposition methods have shown promise in achieving precise stoichiometric control and reducing processing temperatures for BiFeO_3_ films. This opens the door to the use of inexpensive, temperature-sensitive flexible substrates such as plastics. On the other hand, CSD methods also enable uniform coatings over large areas, facilitating the incorporation of BiFeO_3_ films into consumable and wearable electronics, the cornerstone of the internet of things (IoT) (Figure 22). Here, the performance requirements are less stringent compared to the aforementioned microelectronic devices fabricated on single-crystal semiconductor substrates.

Furthermore, the relatively low bandgap of BiFeO_3_-based materials (≤2.8 eV) makes these thin films highly attractive for photovoltaic and photocatalytic applications, such as light-harvesting self-powered devices or cost-effective remediation systems. This broadens the scope of BiFeO_3_ films beyond digital information, opening possibilities in the fields of energy and environment (Figure 22). Large-area solution deposition on affordable, lightweight plastic substrates further enhances the potential of BiFeO_3_-perovskite layers in these fields, offering significant advantages due to their stability in harsh environments (e.g., moisture, radiation, temperature).

However, for solution-deposited BiFeO_3_ films on both rigid single-crystal and flexible substrates, there are still challenges that need to be addressed in order to successfully fabricate devices applicable to advanced technologies. Some of these challenges are associated with the type of device and the three different areas of application considered in this spotlight article: digital information, energy, and environment. These challenges are correlated with the required physical properties for use in these devices, as summarized in Table 1.

**Table 1:** Solution-Deposited Ferroelectric BiFeO_3_ Perovskite-Based Films, Highlighting in the Table Some of the Relevant Properties Sought by Emerging Technologies to Address Current Societal Challenges^a^

field of application	technology	relevant bifeo3-based film properties related to the application	challenging bifeo3-based film requirements
digital transformation (sustainable electronics, internet of things-IoT)	post-silicon electronics	primarily associated with the multiferroic behavior polarization switching piezoelectricity magnetism magnetoelectricity	films on alternative substrates (metal foils, flexible inorganic substrates such as mica, and polymers that should be directed toward biobased polymers) low c-footprint CSD processing methods recyclability biocompatibility
digital transformation (advanced computational method)	memristive memories neuromorphic computing	primarily associated with the multiferroic behavior polarization switching capacitor resistor inductor memristor	epitaxial/highly oriented films on nonsingle-crystal substrates toward cost-effective film deposition methods, avoiding expensive and contaminating photolithography techniques toward inexpensive flexible devices recyclability biocompatibility
digital transformation (autonomous, inexpensive, lightweight devices)& sustainability and energy (low energy consumption electronics)	self-powered electronic devices wireless sensor networks	primarily associated with the multiferroic and photoferroic behavior,which enable energy harvesting for the devices power supply piezoelectricity magnetoelectricity Photovoltaic effect	toward flexible/inexpensive biobased polymer substrates low c-footprint CSD processing methods synergy of current low-temperature solution deposition methods to overcome the barrier of temperatures below 200 °C recyclability biocompatibility
sustainability and energy (pollutants degradation, photocatalytic water splitting, photovoltaics)	photocatalytic remediation solar-to-chemical conversion solar-to-electrical conversion	primarily associated with the ferroelectric and photoferroic behavior piezocatalysis photocatalysis photovoltaic effect	large-area film deposition on inexpensive,nontoxic,and lightweight substrates low c-footprint CSD processing methods synergy of current low-temperature solution deposition methods to overcome the barrier of temperatures below 200 °C Development of large-area electric polarization methods(e.g.corona poling) recyclability biocompatibility

a Additionally, the material requirements that remain challenging for these films are also indicated.

For epitaxial films on rigid single-crystal substrates, despite the environmentally friendly, low-carbon footprint of the low-temperature solution deposition methods used for nontoxic BiFeO_3_ films, the expensive and contaminating microfabrication techniques required to achieve high-performance miniaturized devices for advanced computation unfortunately cannot be avoided at present.

In the case of devices for flexible electronics, future research should focus on the use of biobased flexible polymer substrates, which would enable the fabrication of biocompatible devices. Due to their low degradation temperatures, these types of polymers require the synergy of already developed low-temperature solution deposition methods to reduce the crystallization temperature of the BiFeO_3_ perovskite film, directly deposited on them, to near 200 °C. This is an extremely challenging task in the field of metal oxide films. The recyclability of these biocompatible flexible electronic materials is another issue that needs to be addressed in the near future. For flexible BiFeO_3_ films, emphasis should also be placed on the development of characterization methods capable of assessing the performance of flexible electronic devices under in-operando conditions (e.g., during flexing, stretching, etc.).

Efforts should also be directed toward the development of self-powered flexible electronic devices that can utilize the piezoelectricity, magnetoelectricity, or photovoltaic effect of BiFeO_3_-based perovskite for energy harvesting, which would make it possible to develop wireless sensor networks for implementation in remote environments.

Regarding future applications in the fields of Energy and Environment for large-area solution-deposited BiFeO_3_ films on inexpensive, nontoxic substrates, research into biobased polymer substrates should be emphasized, along with the development of recyclability methods. These are key factors in using these materials in technologies such as remediation and solar-to-chemical conversion, which rely on the photocatalytic activity of the BiFeO_3_ perovskite, or solar-to-electrical conversion, utilizing the photovoltaic effect of BiFeO_3_. However, for these applications, it is not only necessary to deposit BiFeO_3_ films over a large area of an inexpensive, nontoxic substrate but also to perform ferroelectric poling over the entire device. This would allow for the exploitation of the bulk photovoltaic effect, a characteristic of low-bandgap ferroelectrics such as BiFeO_3_. This would enhance charge carrier transfer and suppress recombination, leading to polarization-enhanced photovoltaic and photocatalytic activity in these large-area deposited films. Therefore, the development of corona poling techniques or other novel methods for the electric polarization of large film areas is essential for implementing BiFeO_3_ film devices in energy and environment applications.

All of these advancements in the investigation of BiFeO_3_-based perovskite films would unlock the multifunctional capabilities of these materials and enable their successful use in upcoming advanced devices.

## References

[ref1] Catalan G.; Scott J. F. Physics and Applications of Bismuth Ferrite. Adv. Mater. 2009, 21, 2463–2485. 10.1002/adma.200802849.

[ref2] Wu J.; Fan Z.; Xiao D.; Zhu J.; Wang J. Multiferroic bismuth ferrite-based materials for multifunctional applications: Ceramic bulks, thin films and nanostructures. Prog. Mater. Sci. 2016, 84, 335–402. 10.1016/j.pmatsci.2016.09.001.

[ref3] Wang J.; Neaton J. B.; Zheng H.; Nagarajan V.; Ogale S. B.; Liu B.; Viehland D.; Vaithyanathan V.; Schlom D. G.; Waghmare U. V.; Spaldin N. A.; Rabe K. B.; Wuttig M.; Ramesh R. Epitaxial BiFeO_3_ Multiferroic Thin Film Heterostructures. Science 2003, 299 (5613), 1719–1722. 10.1126/science.1080615. 12637741

[ref4] Lebeugle D.; Colson D.; Forget A.; Viret M. Very large spontaneous electric polarization in BiFeO_3_ single crystals at room temperature and its evolution under cycling fields. Appl. Phys. Lett. 2007, 91 (2), 022907 10.1063/1.2753390.

[ref5] Ramesh R.; Spaldin N. A. Multiferroics: progress and prospects in thin films. Nat. Mater. 2007, 6, 21–29. 10.1038/nmat1805. 17199122

[ref6] Paillard C.; Bai X. F.; Infante I. C.; Guennou M.; Geneste G.; Alexe M.; Kreisel J.; Dkhil B. Photovoltaics with ferroelectrics: current status and beyond. Adv. Mater. 2016, 28 (26), 5153–5168. 10.1002/adma.201505215. 27135419

[ref7] Nechache R.; Harnagea C.; Li S.; Cardenas L.; Huang W.; Chakrabartty J.; Rosei F. Bandgap tuning of multiferroic oxide solar cells. Nat. Photonics 2015, 9, 61–67. 10.1038/nphoton.2014.255.

[ref8] Jain A.; Wang Y. G.; Kumar A.; Gupta N.; Kumar K.; Goyal A. K. BiFeO_3_-based lead-free materials: Recent breakthroughs and their multifunctional applications. J. Alloys Compd. 2025, 1010, 177170 10.1016/j.jallcom.2024.177170.

[ref9] Sun B.; Zhou G.; Sun L.; Zhao H.; Chen Y.; Yang F.; Zhao Y.; Song Q. ABO_3_ multiferroic perovskite materials for memristive memory and neuromorphic computing. Nanoscale Horiz. 2021, 6, 939–970. 10.1039/D1NH00292A. 34652346

[ref10] Seyfouri M. M.; Wang D. Recent progress in bismuth ferrite-based thin films as a promising photovoltaic material. Crit. Rev. Solid State Mater. Sci. 2021, 46 (2), 83–108. 10.1080/10408436.2019.1708700.

[ref11] Lam S.-M.; Sin J.-C.; Mohamed A. R. A newly emerging visible light-responsive BiFeO_3_ perovskite for photocatalytic applications: A mini review. Mater. Res. Bull. 2017, 90, 15–30. 10.1016/j.materresbull.2016.12.052.

[ref12] Liu Y.; Yang B.; He H.; Yang S.; Duan X.; Wang S. Bismuth-based complex oxides for photocatalytic applications in environmental remediation and water splitting: A review. Sci. Total Environ. 2022, 804, 150215 10.1016/j.scitotenv.2021.150215. 34798743

[ref13] Wang N.; Luo X.; Han L.; Zhang Z.; Zhang R.; Olin H.; Yang Y. Structure, Performance, and Application of BiFeO_3_ Nanomaterials. Nano-Micro Lett. 2020, 12, 81 10.1007/s40820-020-00420-6. PMC7770668 34138095

[ref14] Han X.; Ji Y.; Yang Y. Ferroelectric Photovoltaic Materials and Devices. Adv. Funct. Mater. 2022, 32, 2109625 10.1002/adfm.202109625.

[ref15] Wu L.; Ji Y.; Dan H.; Bowen C. R.; Yang Y. A multifunctional optical-thermal logic gate sensor array based on ferroelectric BiFeO_3_ thin films. InfoMat 2023, 5, e12414 10.1002/inf2.12414.

[ref16] Rieck J. L.; Cipollini D.; Salverda M.; Quinteros C. P.; Schomaker L. R. B.; Noheda B. Ferroelastic Domain Walls in BiFeO_3_ as Memristive Networks. Adv. Intell. Syst. 2023, 5, 2200292 10.1002/aisy.202200292.

[ref17] Li J. F.; Wang J. L.; Wuttig M.; Ramesh R.; Wang N.; Ruette B.; Pyatakov A. P.; Zvezdin A. K.; Viehland D. Dramatically enhanced polarization in (0 0 1), (1 0 1), and (1 1 1) BiFeO_3_ thin films due to epitiaxial-induced transitions. Appl. Phys. Lett. 2004, 84 (25), 5261–5263. 10.1063/1.1764944.

[ref18] Gutierrez-Lazaro C.; Bretos I.; Jimenez R.; Ricote J.; Hosiny H. E.; Perez-Mezcua D.; Jimenez Rioboo R. J.; Garcia-Hernandez M.; Calzada M. L. Solution Synthesis of BiFeO_3_ Thin Films onto Silicon Substrates with Ferroelectric, Magnetic, and Optical Functionalities. J. Am. Ceram. Soc. 2013, 96 (10), 3061–3069. 10.1111/jace.12569.

[ref19] Moniz S. J. A.; Quesada-Cabrera R.; Blackman C. S.; Tang J.; Southern P.; Weaver P. M.; Carmalt C. J.; Carmalt C. J. A simple, low-cost CVD route to thin films of BiFeO_3_ for efficient water photo-oxidation. J. Mater. Chem. A 2014, 2 (9), 2922–2927. 10.1039/c3ta14824f.

[ref20] Singh A.; Khan Z. R.; Vilarinho P. M.; Gupta V.; Katiyar R. S. Influence of thickness on optical and structural properties of BiFeO_3_ thin films: PLD grown. Mater. Res. Bull. 2014, 49, 531–536. 10.1016/j.materresbull.2013.08.050.

[ref21] Zhou Z.; Trassin M.; Gao Y.; Gao Y.; Qiu D.; Ashraf K.; Nan T.; Yang X.; Bowden S. R.; Pierce D. T.; et al. Probing electric field control of magnetism using ferromagnetic resonance. Nat. Commun. 2015, 6, 6082 10.1038/ncomms7082. 25631924

[ref22] Bretos I.; Diodati S.; Jiménez R.; Tajoli F.; Ricote J.; Bragaggia G.; Franca M.; Calzada M. L.; Gross S. Low-Temperature Solution Crystallization of Nanostructured Oxides and Thin Films. Chem. - Eur. J. 2020, 26 (42), 9157–9179. 10.1002/chem.202084266. 32212279

[ref23] Martın-Arbella N.; Bretos I.; Jimenez R.; Calzada M. L.; Sirera R. Metal complexes with N-methyldiethanolamine as new photosensitive precursors for the low-temperature preparation of ferroelectric thin films. J. Mater. Chem. 2011, 21, 9051–9059. 10.1039/c1jm10846h.

[ref24] De Dobbelaere C.; Calzada M. L.; Jimenez R.; Ricote J.; Bretos I.; Mullens J.; Hardy A.; Van Bael M. K. Aqueous Solutions for Low-Temperature Photoannealing of Functional Oxide Films: Reaching the 400 °C Si-Technology Integration Barrier. J. Am. Chem. Soc. 2011, 133 (33), 12922–12925. 10.1021/ja203553n. 21806022

[ref25] Bretos I.; Jiménez R.; Ricote J.; Calzada M. L. Low-temperature crystallization of solution-derived metal oxide thin films assisted by chemical processes. Chem. Soc. Rev. 2018, 47 (2), 291–308. 10.1039/C6CS00917D. 29165444

[ref26] Gumiel C.; Jardiel T.; Villalpando A. P.; Lamotte D.; Calatayud D. G.; Calzada M. L.; Jimenez R.; García-Hernandez M.; Mompean F. J.; Caballero A. C.; Villegas M.; Peiteado M. Suppressing the non-switching contribution in BiFeO_3_-Bi_4_Ti_3_O_12_ based thin film composites to produce room-temperature multiferroic behavior. J. Eur. Ceram. Soc. 2022, 42, 5615–5623. 10.1016/j.jeurceramsoc.2022.06.004.

[ref27] Xu W.; Li H.; Xu J. B.; Wang L. Recent advances of solution processed metal oxide thin-film transistors. ACS Appl. Mater. Interfaces 2018, 10, 25878–25901. 10.1021/acsami.7b16010. 29509395

[ref28] Bretos I.; Jiménez R.; Ricote J.; Rivas A. Y.; Echániz-Cintora M.; Sirera R.; Calzada M. L. Metal complexes with N-methyldiethanolamine as new photosensitive precursors for the low-temperature preparation of ferroelectric thin films. J. Sol-Gel Sci. Technol. 2023, 107, 269–277. 10.1007/s10971-023-06065-2.

[ref29] The Materials 2030 Roadmap. Advanced Materials Initiative AMi2030, https://www.ami2030.eu/roadmap/.

[ref30] Sustainable Development Goals | United Nations Development Programme. https://www.undp.org/sustainable-development-goals.

[ref31] Mohan R. Green bismuth. Nat. Chem. 2010, 2, 336 10.1038/nchem.609. 21124518

[ref32] Regulation (EU) **2024**/1252 of The European Parliament and of The Council. https://eur-lex.europa.eu/eli/reg/2024/1252/oj.2024.

[ref33] Henckens T. Critical raw materials. Governance of the World’s Mineral Resources; Elsevier Science, 2021; pp 61–70. 10.1016/b978-0-12-823886-8.00023-3.

[ref34] Dang D. H.; Filella M.; Omanovíc D. Technology-critical elements: an emerging and vital resource that requires more in-depth investigation. Arch. Environ. Contam. Toxicol. 2021, 81, 517–520. 10.1007/s00244-021-00892-6. 34655300

[ref35] Schaeffer N.; Passos H.; Billard I.; Papaiconomou N.; Coutinho J. A. P. Recovery of metals from waste electrical and electronic equipment (WEEE) using unconventional solvents based on ionic liquids. Crit. Rev. Environ. Sci. Technol. 2018, 48 (13–15), 859–922. 10.1080/10643389.2018.1477417.

[ref36] Palai R.; Katiyar R. S.; Schmid H.; Tissot P.; Clark S. J.; Robertson J.; Redfern S. A. T.; Catalan G.; Scott J. F. Beta Phase and Gamma-Beta Metal-Insulator Transition in Multiferroic BiFeO_3_. Phys. Rev. B 2008, 77, 014110 10.1103/physrevb.77.014110.

[ref37] Achenbach G. D.; James W. J.; Gerson R. Preparation of Single-Phase Polycrystalline BiFeO_3_. J. Am. Ceram. Soc. 1967, 50, 437–442. 10.1111/j.1151-2916.1967.tb15153.x.

[ref38] Popa M.; Preda S.; Fruth V.; Sedlackova K.; Balazsi C.; Crespo D.; Calderón-Moreno J. M. BiFeO_3_ Films on Steel Substrate by the Citrate Method. Thin Solid Films 2009, 517, 2581–2585. 10.1016/j.tsf.2008.10.030.

[ref39] Kim J. K.; Kim S. S.; Kim W. J.; Bhalla A. S.; Guo R. Enhanced Ferroelectric Properties of Cr-Doped BiFeO_3_ Thin Films Grown by Chemical Solution Deposition. Appl. Phys. Lett. 2006, 88, 132901 10.1063/1.2189453.

[ref40] Selbach S. V.; Einarsrud M. A.; Grande T. On the Thermodynamic Stability of BiFeO_3_. Chem. Mater. 2009, 21, 169–173. 10.1021/cm802607p.

[ref41] Bretos I.; Jiménez R.; Gutiérrez-Lázaro C.; Montero I.; Calzada M. L. Defect-mediated ferroelectric domain depinning of polycrystalline BiFeO_3_ multiferroic thin films. Appl. Phys. Lett. 2014, 104, 092905 10.1063/1.4867703.

[ref42] Perez-Rivero A.; Ricote J.; Bretos I.; García-Hernández M.; Calzada M. L.; Jiménez R. Enhanced ferroelectric and ferromagnetic properties in lead-free multilayer composite films based on ferroelectric (Bi_0.5_Na_0.5_)_0.945_Ba_0.055_TiO_3_ and multiferroic BiFeO_3_. J. Appl. Phys. 2015, 117, 064105 10.1063/1.4908069.

[ref43] Rivas Y. A.; López-Fajardo S.; Jiménez R.; Bretos I.; Ricote J.; Calzada M. L. Efecto del dopaje en láminas delgadas de BiFeO3 sobre sus propiedades ferroeléctricas; LVIII Congreso Nacional de la SECV. Libro de abstracts: Madrid – Spain, 2022

[ref44] Bai Y.; Jantunen H.; Juuti J. Energy harvesting research: the road from single source to multisource. Adv. Mater. 2018, 30 (34), 1707271 10.1002/adma.201707271. 29877037

[ref45] Baettig P.; Ederer C.; Spaldin N. A. First principles study of the multiferroics BiFeO3, Bi_2_FeCrO_6_, and BiCrO_3_: structure, polarization, and magnetic ordering temperature. Phys. Rev. B 2005, 72 (21), 214105 10.1103/PhysRevB.72.214105.

[ref46] Jimenez R.; Ricote J.; Bretos I.; Jimenez Riobóo R. J.; Mompean F.; Ruiz A.; Xie H.; Lira-Cantú M.; Calzada M. L. Stress-mediated solution deposition method to stabilize ferroelectric BiFe_1-x_Cr_x_O_3_ perovskite thin films with narrow bandgaps. J. Eur. Ceram. Soc. 2021, 41, 3404–3415. 10.1016/j.jeurceramsoc.2020.12.042.

[ref47] Palneedi H.; Maurya D.; Geng L. D.; Song H.-C.; Hwang G.-T.; Peddigari M.; Annapureddy V.; Song K.; Oh Y. S.; Yang S.-C.; Wang Y. U.; Priya S.; Ryu J. Enhanced Self-Biased Magnetoelectric Coupling in Laser-Annealed Pb(Zr,Ti)O_3_ Thick Film Deposited on Ni Foil. ACS Appl. Mater. Interfaces 2018, 10, 11018–11025. 10.1021/acsami.7b16706. 29309126

[ref48] Arora D.; Kumar K.; Kaur D. Multifunctionality in Electrically Poled PMN–PT/Ni–Mn–In Multiferroic Heterostructure for Flexible Magnetic Field Sensing and Nonvolatile Memory Applications. ACS Appl. Electron. Mater. 2024, 6, 1959–1970. 10.1021/acsaelm.3c01855.

[ref49] Algueró M.; Zia L.; Jiménez R.; Amorín H.; Bretos I.; Barreto A.; Jaffari G. H.; Rodríguez-Castellón E.; Ramos P.; Calzada M. L. Bulk-like ferroelectricity and magnetoelectric response of low-temperature solution-processed BiFeO_3_–PbTiO_3_ films on Ni for metallic MEMS. APL Energy 2023, 1, 036108 10.1063/5.0172616.

[ref50] Barreto A.; Jiménez R.; Ramos P.; Amorín H.; Bretos I.; Algueró M.; Calzada M. L. Flexible high-sensitivity magnetoelectric thin film composites of solution-derived BiFeO_3_-PbTiO_3_ layers on Ni foils. Adv. Electron. Mater., under revision.

[ref51] Nathan A.; Ahnood A.; Cole M. T.; Lee S.; Suzuki Y.; Hiralal P.; Bonaccorso F.; Hasan T.; Garcia-Gancedo L.; Dyadyusha A.; Haque S.; Andrew P.; Hofmann S.; Moultrie J.; Chu D. P.; Flewitt A. J.; Ferrari A. C.; Kelly M. J.; Robertson J.; Amaratunga G. A. J.; Milne W. I. Flexible Electronics: The Next Ubiquitous Platform. IEEE Proc. 2012, 100, 1486–1517. 10.1109/jproc.2012.2190168.

[ref52] Nomura K.; Ohta H.; Takagi A.; Kamiya T.; Hirano M.; Hosono H. Room-temperature fabrication of transparent flexible thin-film transistors using amorphous oxide semiconductors. Nature 2004, 432 (7016), 488–492. 10.1038/nature03090. 15565150

[ref53] Bretos I.; Jiménez R.; Ricote J.; Calzada M. L. Low-Temperature Solution Approaches for the Potential Integration of Ferroelectric Oxide Films in Flexible Electronics. IEEE Trans. Ultrason. Ferroelectr. Freq. Control 2020, 67 (10), 1967–1979. 10.1109/TUFFC.2020.2995287. 32746158

[ref54] Bretos I.; Jiménez R.; Pérez-Mezcua D.; Salazar N.; Ricote J.; Calzada M. L. Low-Temperature Liquid Precursors of Crystalline Metal Oxides Assisted by Heterogeneous Photocatalysis. Adv. Mater. 2015, 27 (16), 2608–2613. 10.1002/adma.201405857. 25776728

[ref55] Bretos I.; Jiménez R.; Wu A.; Kingon A. I.; Vilarinho P. M.; Calzada M. L. Activated Solutions Enabling Low-Temperature Processing of Functional Ferroelectric Oxides for Flexible Electronics. Adv. Mater. 2014, 26, 1405–1409. 10.1002/adma.201304308. 24339131

[ref56] Barrios O.; Jiménez R.; Ricote J.; Tartaj P.; Calzada M. L.; Bretos I. A Sustainable Self-Induced Solution Seeding Approach for Multipurpose BiFeO_3_ Active Layers in Flexible Electronic Devices. Adv. Funct. Mater. 2022, 32, 2112944 10.1002/adfm.202112944.

[ref57] Bretos I.; Jimenez R.; Ricote J.; Calzada M. L. Photochemistry in the Low-Temperature Processing of Metal Oxide Thin Films by Solution Methods. Chem. - Eur. J. 2020, 26, 9277–9291. 10.1002/chem.202000244. 32155291 PMC7496836

[ref58] Bretos I.; Jiménez R.; Ricote J.; Sirera R.; Calzada M. L. Photoferroelectric Thin Films for Flexible Systems by a Three-in-One Solution-Based Approach. Adv. Funct. Mater. 2020, 30, 2001897 10.1002/adfm.202001897.

[ref59] Gomez-Lopez A.; Rivas Y. A.; López-Fajardo S.; Jimenez R.; Ricote J.; Pecharromán C.; Montero I.; Bretos I.; Calzada M. L. *In situ* photogenerated hydroxyl radicals in the reaction atmosphere for the accelerated crystallization of solution-processed functional metal oxide thin films. J. Mater. Chem. C 2023, 11, 2619–2629. 10.1039/D2TC05447G.

[ref60] International Roadmap for Devices and Systems (IRDS), 2024. https://irds.ieee.org/editions/2024. 10.1039/d3na01148h PMC11059534 38694454

[ref61] Tomczyk M.; Bretos I.; Jiménez R.; Mahajan A.; Ramana E. V.; Calzada M. L.; Vilarinho P. M. Direct fabrication of BiFeO_3_ thin films on polyimide substrates for flexible electronics. J. Mater. Chem. C 2017, 5 (47), 12529–12537. 10.1039/c7tc04571a.

